# Ambient RNAs removal of cortex-specific snRNA-seq reveals *Apoe*^+^ microglia/macrophage after deeper cerebral hypoperfusion in mice

**DOI:** 10.1186/s12974-023-02831-9

**Published:** 2023-06-26

**Authors:** Yuan Zhang, Jinyun Tan, Kai Yang, Weijian Fan, Bo Yu, Weihao Shi

**Affiliations:** 1 Department of Vascular Surgery, Shanghai Pudong Hospital, Fudan University Pudong Medical Center, Shanghai, People’s Republic of China; 2 Fudan Zhangjiang Institute, Shanghai, 201203 China; 3 Department of Vascular Surgery, Huashan Hospital of Fudan University, Shanghai, People’s Republic of China

**Keywords:** Ambient RNAs contamination, Deeper chronic cerebral hypoperfusion, Neuroinflammation, *Apoe*^+^ MG/Mac

## Abstract

**Background:**

Ambient RNAs contamination in single-nuclei RNA sequencing (snRNA-seq) is a challenging problem, but the consequences of ambient RNAs contamination of damaged and/or diseased tissues are poorly understood. Cognitive impairments and white/gray matter injuries are characteristic of deeper cerebral hypoperfusion mouse models induced by bilateral carotid artery stenosis (BCAS), but the molecular mechanisms still need to be further explored. More importantly, the BCAS mice can also offer an excellent model to examine the signatures of ambient RNAs contamination in damaged tissues when performing snRNA-seq.

**Methods:**

After the sham and BCAS mice were established, cortex-specific single-nuclei libraries were constructed. Single-nuclei transcriptomes were described informatically by the R package Seurat, and ambient RNA markers of were identified in each library. Then, after removing ambient RNAs in each sample using the in silico approaches, the combination of CellBender and subcluster cleaning, single-nuclei transcriptomes were reconstructed. Next, the comparison of ambient RNA contamination was performed using irGSEA analysis before and after the in silico approaches. Finally, further bioinformatic analyses were performed.

**Results:**

The ambient RNAs are more predominant in the BCAS group than the sham group. The contamination mainly originated from damaged neuronal nuclei, but could be reduced largely using the in silico approaches. The integrative analysis of cortex-specific snRNA-seq data and the published bulk transcriptome revealed that microglia and other immune cells were the primary effectors. In the sequential microglia/immune subgroups analysis, the subgroup of *Apoe*^+^ MG/Mac (microglia/macrophages) was identified. Interestingly, this subgroup mainly participated in the pathways of lipid metabolism, associated with the phagocytosis of cell debris.

**Conclusions:**

Taken together, our current study unravels the features of ambient RNAs in snRNA-seq datasets under diseased conditions, and the in silico approaches can effectively eliminate the incorrected cell annotation and following misleading analysis. In the future, snRNA-seq data analysis should be carefully revisited, and ambient RNAs removal needs to be taken into consideration, especially for those diseased tissues. To our best knowledge, our study also offers the first cortex-specific snRNA-seq data of deeper cerebral hypoperfusion, which provides with novel therapeutic targets.

**Supplementary Information:**

The online version contains supplementary material available at 10.1186/s12974-023-02831-9.

## Introduction

In contrast to traditional bulk RNA sequencing (RNA-seq), single-cell RNA sequencing (scRNA-seq) offers numerous benefits to explore the cell-to-cell heterogeneity of animal models or clinical tissues. However, the dissociation of individual cells from brain tissues presents a challenge [1, 2]. Thus, single-nuclei RNA sequencing (snRNA-seq) has recently emerged as a novel alternative technology for investigating transcriptional activity at single-nucleus resolution. On the one hand, the acquisition of fresh human brain tissue for profiling analysis presents a challenging task. In such circumstances, frozen brain materials are deemed appropriate for snRNA-seq, but not for scRNA-seq. On the other hand, accumulating evidence has shown concordance between snRNA-seq data and scRNA-seq, despite the former solely capturing nuclear mRNA [1, 3, 4]. Both scRNA-seq and snRNA-seq are promising methodologies to elucidate the underlying pathophysiology of diseases. And, the successful generation of high-quality transcriptome maps at single cell/nucleus resolution is a crucial prerequisite for subsequent analyses.

However, there still exist lots of problems to be tackled in snRNA-seq experiments. During the capture of individual nuclei by oil droplets following single-nucleus suspension preparation, transcripts from other cells/nuclei in the suspension are inadvertently captured and attributed to the captured cell/nucleus. The freely floating transcripts from lysed cells/nuclei are known as ambient RNA [5, 6]. Recently, in order to remove “empty droplets” containing ambient RNAs contamination from real nuclei/cells, several developed software tools have been developed, including DecontX, SoupX and CellBender [6–8]. According to Emre Caglayan’s study, CellBender exhibits the highest efficiency in removing ambient RNAs contamination of brain tissues among the mentioned tools [9]. Additionally, Emre Caglayan et al. [9] also found that the problem of ambient RNA contamination produced in the process of snRNA-seq library construction can be partially or completely solved by fluorescence-activated cell sorting (FACS) technique or subsequent in silico methods, such as combining CellBender with sequential subcluster cleaning. However, in their study, the removal of ambient RNAs was performed in healthy brain tissues without pathological changes [9], and the decontamination efficiency of brain tissues under pathological changes has not been further explored. Given that many tissues encountered in preclinical and clinical studies exhibit pathological and/or damaged cells, it becomes more useful and practical to find an effective way to remove ambient RNAs contamination in diseased conditions.

Carotid artery stenosis is a common reason in the aging population to contribute dementia and cognitive deficits [10–12]. Most studies in the field have primarily focused on subcortical white matter lesions of chronic cerebral hypoperfusion induced by carotid artery stenosis [13]. However, clinical studies have indicated a correlation between the presence of cortical cerebral microinfarcts (CMIs) and poor cognitive function in patients with carotid artery stenosis [14]. This suggests that the cortical dysfunction plays an important role in carotid artery stenosis, and more attention should be given to cortical damages. Nonetheless, the bilateral carotid artery stenosis (BCAS) model induced by bilateral 0.18-mm-diameter microcoils fails to induce grey matter lesions. Furthermore, from the aspects of cerebral hemodynamics, bilateral 0.18 mm diameter microcoils fail to lead to the consistent reduction of cerebral blood flow (CBF) [15, 16]. Additionally, most patients usually exhibit asymmetric stenosis of the carotid artery [17, 18]. Based on our previous findings [19, 20] and corroborating studies [21, 22], the BCAS mouse model induced by the 0.16/0.18 mm diameter microcoils can faithfully simulate clinical situations of carotid artery stenosis by consistently reducing CBF, inducing cortical damages, and resulting in cognitive deficits. Thus, we propose that the 0.16/0.18 mm BCAS model is a more appropriate alternative for studying cortical damages and the underlying molecular mechanisms of deeper cerebral hypoperfusion induced by carotid artery stenosis. In the BCAS mouse models (0.16/0.18 mm), there exists not only glial cell activation and peripheral immune cell infiltration, but also a spectrum of neurons in healthy, “sub-healthy”, and diseased conditions, encompassing different cell types in different states [19, 20]. Thus, the neural tissue in this diseased state presents an ideal model to assess the decontamination efficiency of snRNA-seq, due to its high cellular heterogeneity and complexity. In diseased conditions, ambient RNAs can originate not only from cytoplasmic transcripts, but also from nuclei with incomplete nuclear membranes caused by manual operations and/or damaged tissue. Therefore, we hypothesize that more ambient RNA profiles in the disease situation could be found in both empty droplets containing neuronal reads as well as distinctive neuronal ambient RNAs in non-neuronal cell types compared to normal conditions. This potential discrepancy might lead to misinterpretations in the downstream analyses.

Here, we established the mice models with chronic deeper cerebral hypoperfusion and sham operation, as previously described [19, 20], followed by the construction of cortex-specific snRNA-seq. According to the intronic read ratio of ambient RNAs, we categorized them into two types within each individual sample: nuclear ambient RNAs with high intronic read ratios and non-nuclear ones with low intronic read ratios. Then, we identified the sources of cell types and cell components for the ambient RNAs in each sample. By using the in silico methods, specifically CellBender with sequential subcluster cleaning, we constructed four cortex-specific snRNA-seq datasets of high quality for each individual sample. Besides, we assessed the changes of the ambient RNAs contamination of each sample before and after their removal in each individual sample. Furthermore, ambient RNA removal could mitigate the manual artifacts and misleading results to a large extend, facilitating the identification of distinct types of microglia/macrophages (MG/Mac) in this diseased situation, including *Apoe*^+^ microglia/macrophages. Taken together, our results provide an in-depth analysis of ambient RNA contamination when performing profiling of brain tissues in diseased situations, and underscore the importance of the removal of ambient RNAs before the downstream analysis. Last but not least, to our best knowledge, this is the first study to describe the cortex-specific transcriptome after chronic deeper cerebral hypoperfusion at single-nucleus resolution, thereby providing with novel therapeutic targets for the chronic cerebral ischemic injury disease.

## Materials and methods

### Experimental animals and the procedures of bilateral carotid artery stenosis

Adult male C57BL/ 6J mice (11 weeks old) were purchased from Beijing Vital River Laboratory Animal Technology. All the mice were housed in IVCs (Individually Ventilated Cages) under SPF (specific pathogen free) conditions, with sterile water and food ad libitum on a 12-h dark/light cycle. After one week acclimation, all the mice were randomly divided into two groups, the sham operation and BCAS groups. The BCAS procedure was performed as previously described in a previous study [19, 20]. Anesthesia was induced using a higher dose of isoflurane and maintained with 2% isoflurane delivered in medical oxygen. After the successful establishment of anesthesia, the neck skin of the mice was disinfected and cut at the midline. Then, both common carotid arteries (CCAs) were dissected free from surrounding tissues, separated and exposed carefully from vagus nerve by using blunt forceps. Next, two microcoils with inner diameters of 0.16 mm and 0.18 mm were used for the surgical procedures. The 0.16-mm microcoil was firstly twined around the right CCA just below the carotid bifurcation. After one hour, the second microcoil (0.18 mm) was wrapped around the left CCA. The sham group underwent the same surgical procedures, but the microcoils were not applied.

### Cortex-specific single-nuclei library preparation

At the three weeks after sham or BCAS operation, all the mice (four mice in each group) were rapidly decapitated and brain were quickly removed. As described in our previous study, the right cortex (0.16 mm side) in the BCAS group exhibited more severe brain damages, characterized by micro-stroke-like neuropathology [19, 20]. Thus, in this study, the two right cortices from two mice in the same group were dissected and pooled together for the further nuclei suspension preparation. After washed by cold 1 × PBS, the brain tissue was transferred into the dounce homogenizer containing 5 ml of lysis buffer (0.32 M sucrose, 5 mM CaCl2, 3 mM Mg(Ace)_2_, 0.1 mM EDTA, 10 mM Tris–HCl pH = 8, 0.1% NP-40) containing 0.2 U/μl RNase Inhibitor (Thermo EO0382). After 20 times of up-and-down manual strokes on ice, the homogenized sample was transferred into a new 15 ml conical tube, followed by the centrifugation (4000 r.p.m., 4 °C, 5 min). After centrifugation, the supernatant was discarded, and the precipitation (nuclei pellet) was retained. Then, the 15-ml tube was added with 1 ml of lysis buffer, and the nuclei pellet was pipetted by using a sterile 1-ml pipette tip until the nuclei were well distributed. After the nuclei suspension was sequentially added with 4 ml of lysis buffer and 5 ml of sucrose solution (1.8 M sucrose, 3 mM Mg(Ace)_2_, 10 mM Tris–HCl pH = 8), the nuclei suspension was mixed well by gently inverting the tube ten times. After the centrifugation again (4000 r.p.m., 4 °C, 5 min), the supernatant was discarded, and the precipitation (nuclei pellet) was retained. After the nuclei pellet was resuspended in 1 ml of FBS buffer (1 × PBS with 2% FBS, fetal bovine serum) containing 0.2U/μl RNase Inhibitor, the nuclei pellet was pipetted by 1 ml and 200 μl pipette tip until the nuclei were well distributed. After 500 μl of nuclei suspension was taken for being filtered through a 40-μm cell strainer, the nuclei suspension was added 4 ml of FBS buffer. The number of all nuclei of four samples was determined by Countstar. After the first nuclei count, all the nuclei solution was adjusted to a final concentration of 500–1200/μl. All nuclei suspension of four samples showed low background (Additional file 1: Figure S1-S4), and more information about the nuclei suspension is shown in Additional file 2: Table S1. Considering the capture efficiency of 10X Chromium microfluidics system (10X Genomics), we used about 20,000 nuclei for further procedures in order to capture a final number of about 10,000 nuclei. According to the manufacturer’s protocol (10X Genomics #CG00052), the single-nuclei capture, barcoding and library preparation were performed by using the 10X Genomics platform, using version 3 chemistry. The captured nuclei were lysed, and the released RNA of nuclei was barcoded through reverse transcription in individual Gel Bead in emulsion (GEM), and the quality of the reverse transcription products were assessed using an Agilent 4200 (Additional file 1: Figure S5-S6, Additional file 2: Table S2). Then, the cDNA products were amplified, followed by the library construction, and the library quality was assessed using an Agilent 4200 (Additional file 1: Figure S5, S7, Additional file 2: Table S2). Finally, the library was sent to Huada Genomics Co., Ltd for 150 bp paired-end (PE) sequencing.

### Data analysis

**Fig. 1:**
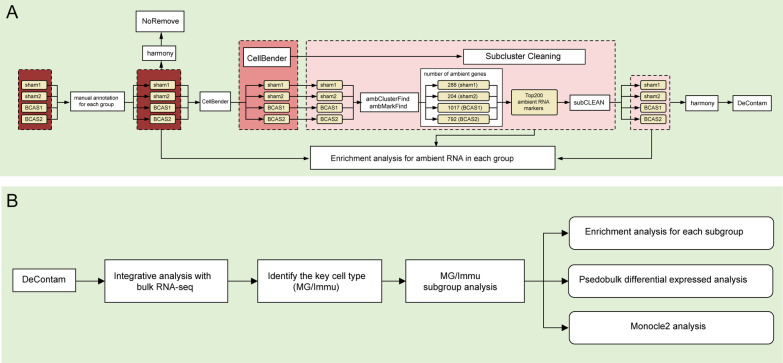
Flowchart of bioinformatic analyses of snRNA-seq. **A** The procedures of ambient RNAs removal. **B** The analysis of snRNA-seq after ambient RNAs contamination removal. NoRemove, snRNA-seq data of four samples before ambient RNAs contamination removal. DeContam, snRNA-seq data of four samples after ambient RNAs contamination removal. MG/Immu, microglia and other immune cell

The software CellRanger toolkit (version 6.1.2) was used to filter low-quality reads, align to a reference mouse genome (mm10), generate a unique molecular identifier (UMI) matrix, and assign cell barcodes [23]. Then, the matrix was further analyzed by the R package Seurat developed by the Satija Lab [24], the software CellBender, some R packages published in Emre Caglayan’s study [9], and others for the downstream analysis. Details of methods were described as follows:

1. The analysis of snRNA-seq data before ambient RNAs removal
  After getting the filtered matrix from CellRanger, *NormalizeData* function in *Seurat* was used to normalize data, *FindVariableFeatures* function was used to identify hypervariable genes, *ScaleData* function was used to centralize data, and *FindNeighbors*, *FindClusters*, and *RunTSNE* were used for dimensions reduction of clustering nuclei in each single sample. Then, the marker genes of each group were manually annotated (the marker genes of neurons include *Snap25*, *Rbfox3* and *Grin2b*; the marker genes of microglia include *Hexb*; the marker genes of astrocytes include *Slc1a2*; the marker genes of oligodendrocytes include *Plp1* and *Mbp*; the marker genes of oligodendrocyte precursor cells include *Lhfpl3*; The marker genes of fibroblasts include A*dam12*; the marker genes of endothelial cells include *Flt1*). Subsequently, the four samples were integrated together by the R package harmony, and named as NoRemove, single-nuclei population without ambient RNAs removal. The characteristic gene set of each group was searched by the *FindAllmaker* function. Besides, the pseudobulk differentially gene expression analysis was performed in the microglia/immune nuclei subset by using the R package muscat (BCAS vs sham) [25].
2. The analysis of ambient cluster and identification of ambient marker genes
  Using the *ambClusterFind* function with by default parameters in the study of Emre Caglayan et al. [9], the unfiltered original expression matrix was used as input data to find the nuclei population with ambient RNAs contamination. Subsequently, the *ambMarkFind* function was used to find ambient marker genes. In this step, the “logfc” was set as 0, and other parameters were set by default.
3. The analysis of snRNA-seq data after ambient RNAs removal
  After the presence of ambient RNAs in the single-nuclei library was found, the ambient RNA contamination was removed in each single-nuclei sample (Fig. 1A). The unfiltered original expression matrix of each sample derived by CellRanger was imported into the software CellBender for the first step of ambient RNAs removal. In this step, the input was the raw gene–cell barcode count matrix, the deposited data in GSE229259. After the CellBender treatment, the additional subcluster cleaning was performed as described previously in Emre Caglayan et al.’s study [9]. In details, the cluster analysis was performed similarly as mentioned above by using the *FindAllmaker* function. Meanwhile, we selected the top 200 (by logfc) ambient RNA markers in each sample. Then, with these two input data, the *subCLEAN* function was used to identify and remove contaminated subsets in each non-neuronal group of each sample (FDR < 0.001 and odds ratio > 3). After two separate rounds of decontamination for each sample, they were integrated using the harmony function and named as DeContam. The following analysis was performed as similarly as the analysis before ambient RNAs removal mentioned above. After that, we also performed the changes of the ambient RNAs enrichment in each sample before and after decontamination by using irGSEA. In this step, we selected the top 200 ambient RNA marker genes mentioned above as input data, and the details of the irGSEA enrichment analysis were shown in our previous study [26].
  After removing the ambient RNAs, further analysis was carried out in the DeContam (Fig. 1B). First of all, our published cortex-specific bulk RNA-seq data of severe carotid stenosis group and sham operation group were downloaded from GEO database (GSE210666). Differential analysis of bulk RNA-seq was performed by using DESeq2 software package. Subsequently, the bulk RNA-seq and snRNA-seq were analyzed jointly using the irGSEA package. After extracting the microglia/immune nuclei subsets, the cluster analysis and dimension reduction were carried out again. In addition, the pseudobulk differentially gene expression analysis was performed in the microglia/immune nuclei subset by using the R package muscat (BCAS vs sham). Meanwhile, the irGSEA enrichment analysis was also performed by using the input data in our study and the published middle cerebral artery occlusion (MCAO) scRNA-seq data [27]. The R package monocle2 was used for the pseudo-time sequence analysis of the microglia/immune nuclei subsets.

### Data availability

The snRNA-seq data used in this study are available at the Gene Expression Omnibus (GEO) with the access number of GSE229259.

## Results

### The misleading results induced by ambient RNAs contamination in snRNA-seq data

**Fig. 2:**
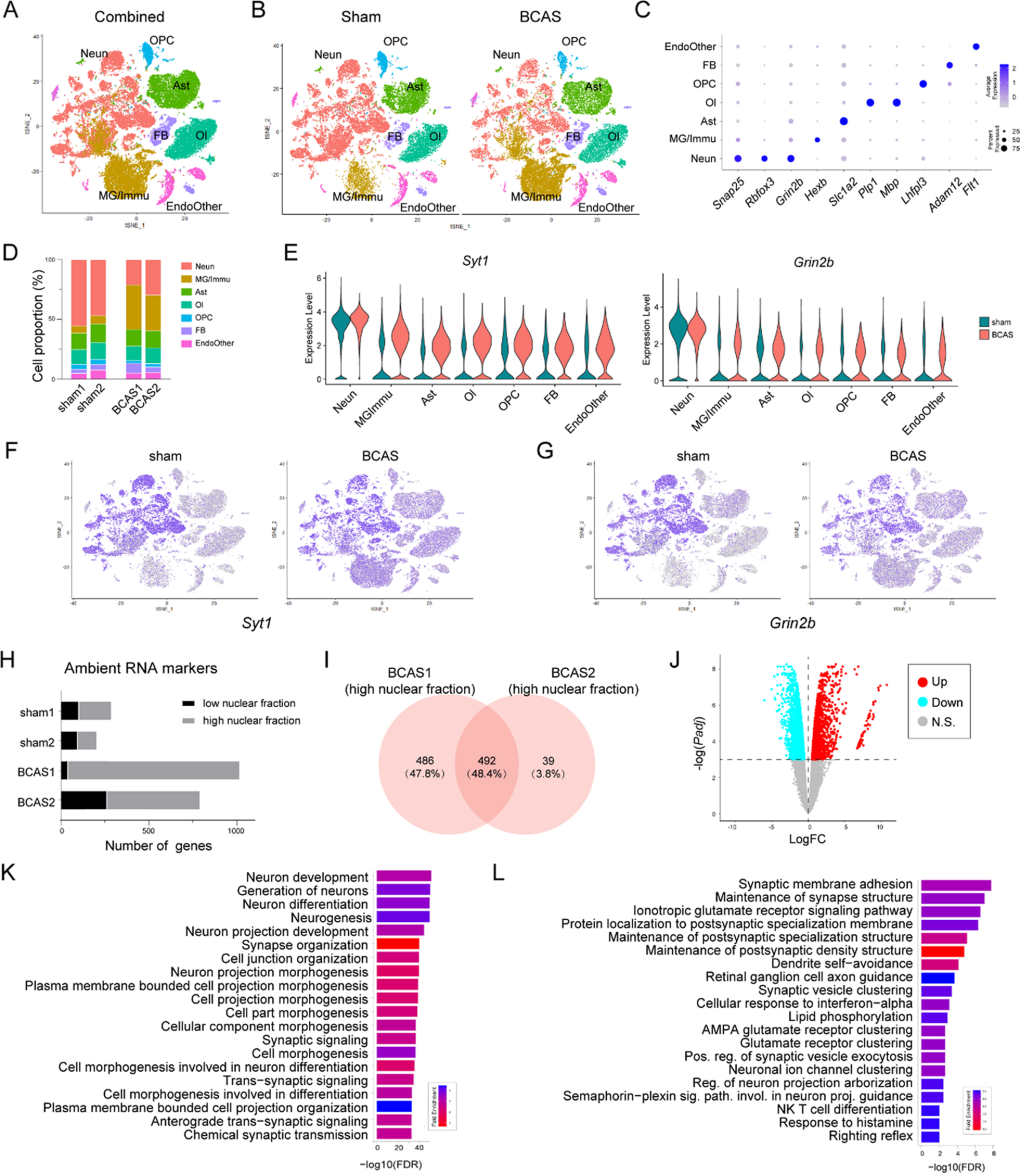
Cortex-specific snRNA-seq datasets of sham/BCAS mice contaminated by ambient RNAs. Neun, neuron; MG/Immu, microglia and other immune cell; Ast, astrocyte; Ol, oligodendrocyte; OPC, oligodendrocyte precursor cell; FB, fibroblast; EndoOther, endothelial cell and other cell. **A** T-distributed stochastic neighbor embedding (t-SNE) plot shows single-nuclei in all combined samples (combined, sham and BCAS groups). **B** Stacked column plots show the cell proportions of in different groups between the sham and BCAS groups. **C** t-SNE plots show all single-nuclei in the sham and BCAS groups, respectively. **D** Dot plots show the average expression of specific genes in different groups. **E** Violin plots show the expression of *Syt1* and *Grin2b* in different groups between the sham and BCAS groups. **F, G** t-SNE plots show the expression of *Syt1* and *Grin2b* in different groups between the sham and BCAS groups. **H** Stacked column plots show nuclear and non-clear ambient RNAs in four samples (sham1, sham2, BCAS1 and BCAS2). **I** Venn plot shows the overlap of nuclear ambient RNA markers between BCAS1 and BCAS2. **J** Volcano plot shows DEGs of microglia between the sham and BCAS groups. **K** Bar plots show the pathway enrichment of the overlap ambient RNA markers in **I**. **L** Bar plots show the pathway enrichment of up-regulated genes of microglia in Panel **J**

The analysis of the snRNA-seq data revealed several crucial findings regarding ambient RNA contamination and its potential impact on the interpretation of results. According to Fig. 2A, although most nuclei subsets could be separated independently, yet some “Neun” and “MG/Immu” subsets were still overlapped. Additionally, we also observed that compared with the samples of the two sham samples, the neuronal proportions in the two samples of BCAS group decreased significantly, while the MG/Immu proportions increased significantly (Fig. 2B). As shown in Fig. 2C, the nuclei with overlapping parts mainly came from neuronal subsets in the sham group and MG/Immu subsets in the BCAS group. As shown in the dot plots (Fig. 2D), although *Snap25*, *Rbfox3*, *Grin2b*, *Hexb*, *Slc1a2*, *Plp1*, *Mbp*, *Lhfpl3*, *Adam12* and *Flt1* were all highly expressed in their corresponding nuclei subsets, yet they were also expressed in other nuclei subsets. As expected, in the snRNA-seq data generated by nuclei isolation but not nuclei sorting (purification of DAPI^+^ nuclei with flow cytometry), the existence of ambient RNA, especially in the non-neuronal groups, is an inevitable issue [9]. Because the gene expression of neurons in CNS is more abundant than that of other cell subtypes, other cell types will be contaminated by neurons. Therefore, we observed the expression of two neuronal marker genes (*Syt1* and *Grin2b*) in each nuclei subset, and found that these two genes were expressed to a certain extent in other nuclei subsets except neuronal subsets in both sham and BCAS groups (Fig. 2E). Moreover, according to the t-SNE plots (Fig. 2F, G), the two genes (*Syt1* and *Grin2b*) in non-neuronal subsets exhibited greater expression than that in sham group. Besides, as shown in the barcode rank plots generated by CellRanger (Additional file 1: Figure S8), we found the UMI counts of the background platform were near 1000 in both BCAS samples, while that were between 100 and 1000 in both sham samples. Thus, we speculated that there might exist more ambient RNA in non-neuronal subsets of BCAS group. To test this speculation, we used the *ambMarkFind* function (logfc = 0) to find ambient RNA markers in each sample. As shown in the bar plots (Fig. 2H), the number of ambient RNA markers in both BCAS samples increased compared with sham group. Moreover, most of the ambient RNA markers in BCAS group were genes in the nuclei, suggesting that because the nuclei in BCAS group were in a state of injury, the transcripts in the nuclei were more likely to release into the nuclei suspension during nuclei extraction, resulting in ambient RNA contamination. We analyzed the enrichment analysis of 492 ambient RNA markers in the nuclei of BCAS1 and BCAS2 samples, and found that they were predominantly associated with neuronal functions, further suggesting that these ambient RNA markers mainly came from the damaged nuclei of BCAS group (Fig. 2I, K). Since both absolute number and transcripts of neurons are more than those in non-neuronal cells in the adult cortex [28], we speculated that there were more ambient RNA contamination in non-neuronal subtypes of BCAS group, which could potentially lead to misleading results in subsequent differential expression analysis (BCAS vs sham). To verify this speculation, we used the R package muscat to perform subsequent pseudobulk differential gene analysis (BCAS vs sham) of MG/Immu subsets, which do not originate from central nervous system (CNS). As shown in the volcano plot (Fig. 1J), out of the 3825 differentially expressed genes (DEGs), 1935 were significantly up-regulated in the BCAS group compared to the sham group (Additional file 2: Table S3). Although the immune response-related pathways were among the 20 most enriched pathways of up-regulated genes in the BCAS group, the majority of these pathways were related to neuronal and synaptic functions. These results indicated that in the process of single-nuclei sequencing, the non-neuronal subsets in the BCAS group exhibited more ambient RNA contamination than that in the sham group. If the ambient RNA contamination was not adequately addressed, it could potentially lead to some misleading results and conclusions.

### The features of nuclei depleted by the decontamination treatment

**Fig. 3:**
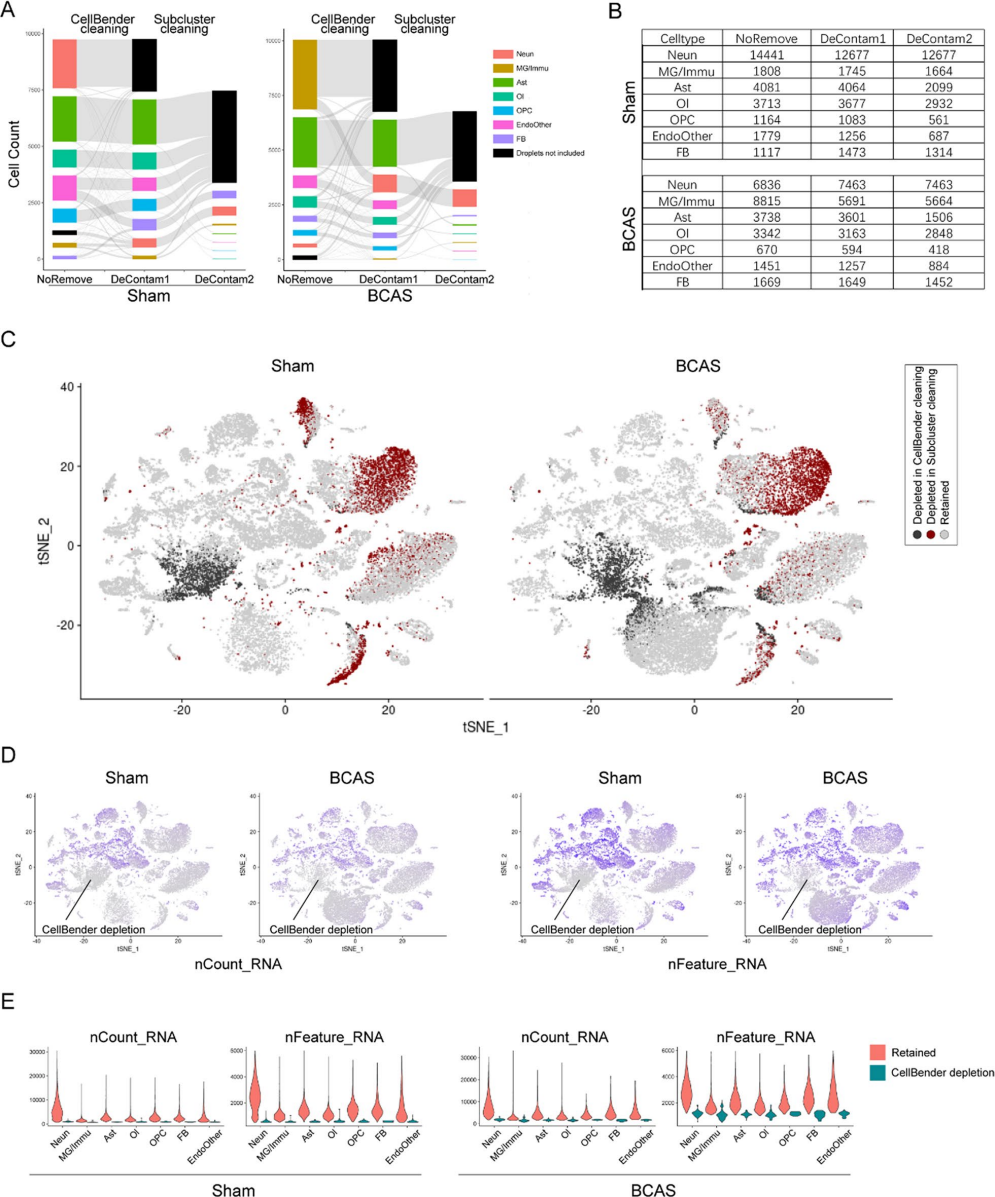
The features of nuclei depleted by the decontamination treatment. Neun, neuron; MG/Immu, microglia and other immune cell; Ast, astrocyte; Ol, oligodendrocyte; OPC, oligodendrocyte precursor cell; FB, fibroblast; EndoOther, endothelial cell and other cell. **A** Sankey plots show the nuclei with changing annotation after the two-step decontamination treatment. **B** Tables show the count of nuclei before and after decontamination treatments in different cell types of the sham and BCAS groups. **C** t-SNE plots show the nuclei depleted in CellBender and subcluster cleaning treatments, respectively. **D** t-SNE plots show the nCount and nFeature of all nuclei. **E** Violin plots show the comparison of the nCount and nFeature in different cell types between NoRemove and CellBender cleaning. Retained, the nuclei retained after CellBender cleaning; CellBender depletion, the nuclei depleted after CellBender cleaning

According to previous literature reports, the combination of CellBender and subcluster cleaning method has been suggested for decontamination of ambient RNAs for single-nuclei sequencing in non-injured brains [9]. Therefore, we tried to test whether we could use this method to effectively remove the ambient RNAs of cortical single-nuclei data obtained from chronic cerebral damages. In Fig. 3A, NoRemove, DeContam1 and DeContam2 were all the *Seurat* objects. In the NoRemove object, “Droplets not included” referred to the droplets initially filtered by CellRanger at but later confirmed as real nuclei after CellBender treatment. In the DeContam1 object, “Droplets not included” indicated droplets considered as nuclei but depleted after CellBender treatment. In the DeContam2 object, “Droplets not included” represented droplets considered as real nuclei with ambient RNAs contamination but depleted after subcluster cleaning treatment. After the CellBender treatment in the first step, the main discarded nuclei population in sham group (Fig. 3A, left) belonged to the subset of “neuronal group”, while in the BCAS group (Fig. 3A, right), the main discarded nuclei population was the subset of “microglial group”. Subsequently, after subcluster cleaning treatment, some parts of each nuclei subgroup in the two groups were discarded (Fig. 3A). The table in Fig. 3B showed the nuclei number in each subset before and after the first step of CellBender treatment, as well as the second step of subcluster decontamination treatment in sham and BCAS groups. Interestingly, among the nuclei removed by CellBender at the first step (Fig. 3C), most of them were overlapping nuclei in the sham and BCAS groups, which were the subsets initially misannotated as “neuronal nuclei” in the sham group and the subsets misannotated as “microglial/immune nuclei” in the BACS group (Fig. 2A). Furthermore, according to the t-SNE plots in Fig. 3D, both the count and nFeature of the nuclei subpopulation removed by CellBender were lower than those retained after CellBender treatment. In other nuclei subsets, similar results were found (Fig. 3E). These results suggested that the “nuclei subsets” depleted by CellBender might be empty droplets that only contained ambient RNA and had no nuclei. However, most of the nuclei subsets removed by subcluster cleaning treatment in the second step were astrocytes in both sham and BCAS groups, and their count and nFeature numbers were similar to those in the retained nuclei subsets (Fig. 3C, D). These results suggested that the droplets which contained both nuclei and ambient RNA might be removed in this step.

Considering that the biggest discarded nuclei populations in the sham and BCAS groups by CellBender were “Neun” and “MG/Immu” groups, respectively (Fig. 3A), we extracted these two discarded nuclei populations for further analysis of the ambient RNAs features in the BCAS group. Interestingly, although these two discarded populations were overlapped in the t-SNE plot, yet the discarded “MG/Immu” population in the BCAS group exhibited enrichment in certain immune-related pathways (Additional file 1: Figure S11 A-B). In the nuclei depleted by CellBender, according to the t-SNE plots (Fig. 2F–G, Additional file 1: Figure S9) and dot plots (Additional file 1: Figure S11C), although the comparable percentages of nuclei expressing neuronal genes were found between the “MG/Immune” population in the BCAS group and the “Neun” population in the sham group, yet the genes *P2ry12* and *Hexb* were also highly expressed in the nuclei of the BCAS group. According to the results mentioned above, in the BCAS group, most ambient RNAs came from neuronal group, accompanied with some dead microglial transcripts.

### Ambient RNAs of non-neuronal population could be removed effectively with in silico methods

**Fig. 4:**
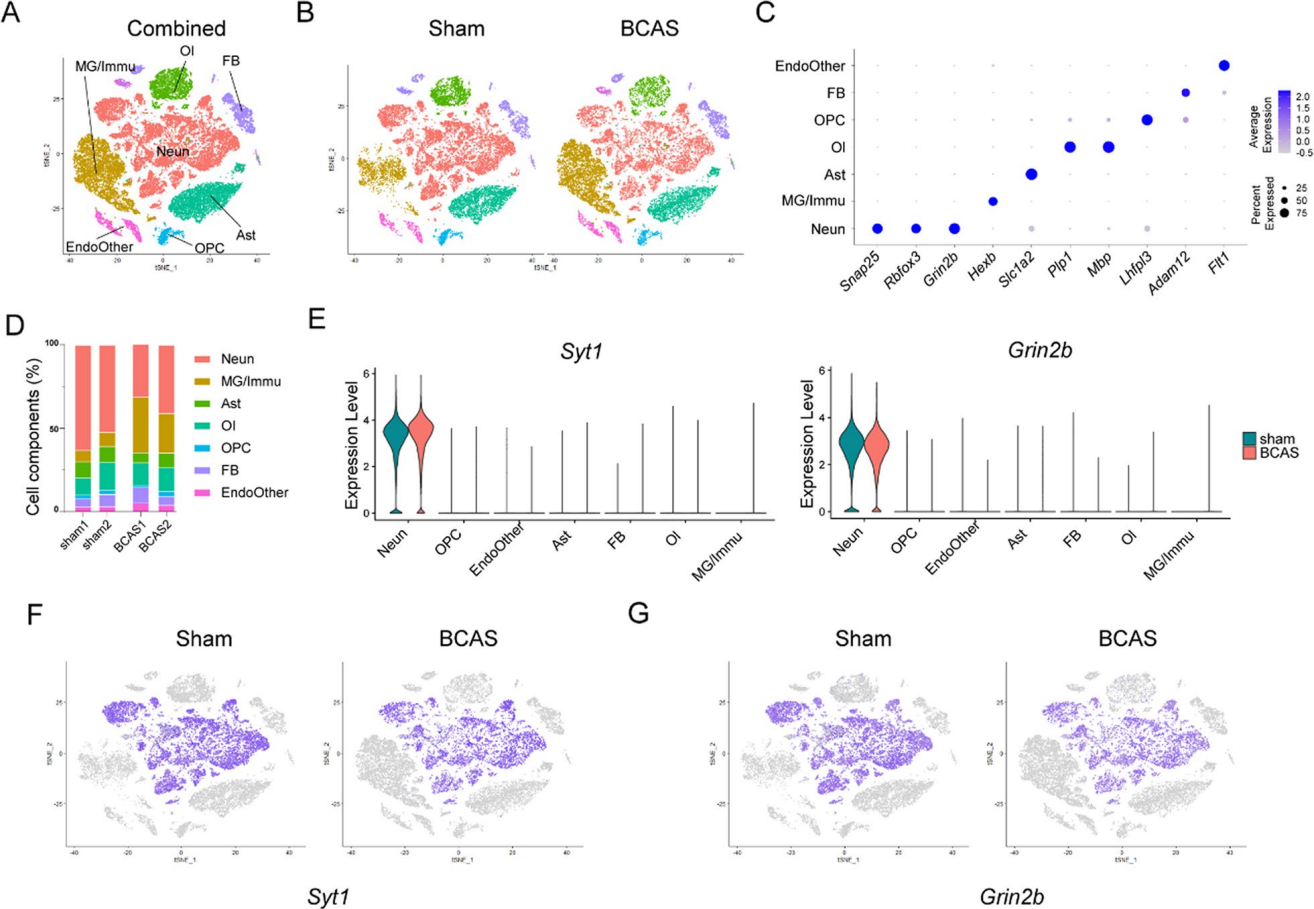
Cortex-specific snRNA-seq datasets of sham/BCAS mice after ambient RNA removal. **A** t-SNE plots show all single-nuclei in the sham and BCAS groups, respectively.** B** t-SNE plots show all single-nuclei in the sham and BCAS groups, respectively. **C** Stacked column plots show the nuclei proportions of in different clusters between the sham and BCAS groups.** D** Dot plots show the average expression of specific genes in different groups. **E** Violin plots show the expression of *Syt1* and *Grin2b* in different clusters between the sham and BCAS groups. **F, G** t-SNE plots show the expression of *Syt1* and *Grin2b* in different clusters between the sham and BCAS groups

After decontamination of ambient RNA in each single-nuclei data by using the combination of CellBender and Subcluster cleaning methods, we combined the four single-nuclei data again by using the harmony function. As shown in Fig. 4A, B, there existed no overlapping nuclei population of different cell types after ambient RNA removal. As shown in the bar plots (Fig. 4C), similar to the results before decontamination, the proportions of neuronal subsets in BCAS group decreased compared with sham group, while the proportions of microglial and other immune subsets increased. Moreover, *Snap25*, *Rbfox3*, *Grin2b*, *Hexb*, *Slc1a2*, *Plp1*, *Mbp*, *Lhfpl3*, *Adam12* and *Flt1* were highly expressed in their corresponding nuclei subsets, but hardly expressed in other subsets (Fig. 4D). Thus, unlike the results before decontamination treatment (Fig. 2C), the signal-to-noise ratios of characteristic genes were higher after decontamination treatment (Fig. 4D). Moreover, after decontamination, *Syt1* and *Grin2b* were exclusively expressed in neuronal subsets, but not in non-neuronal subsets (Fig. 4E–G). Subsequently, we took the top 200 characteristic genes of each subgroup for the further GO analysis (Additional file 1: Figure S12), revealing unique functional pathways for each subset.

**Fig. 5:**
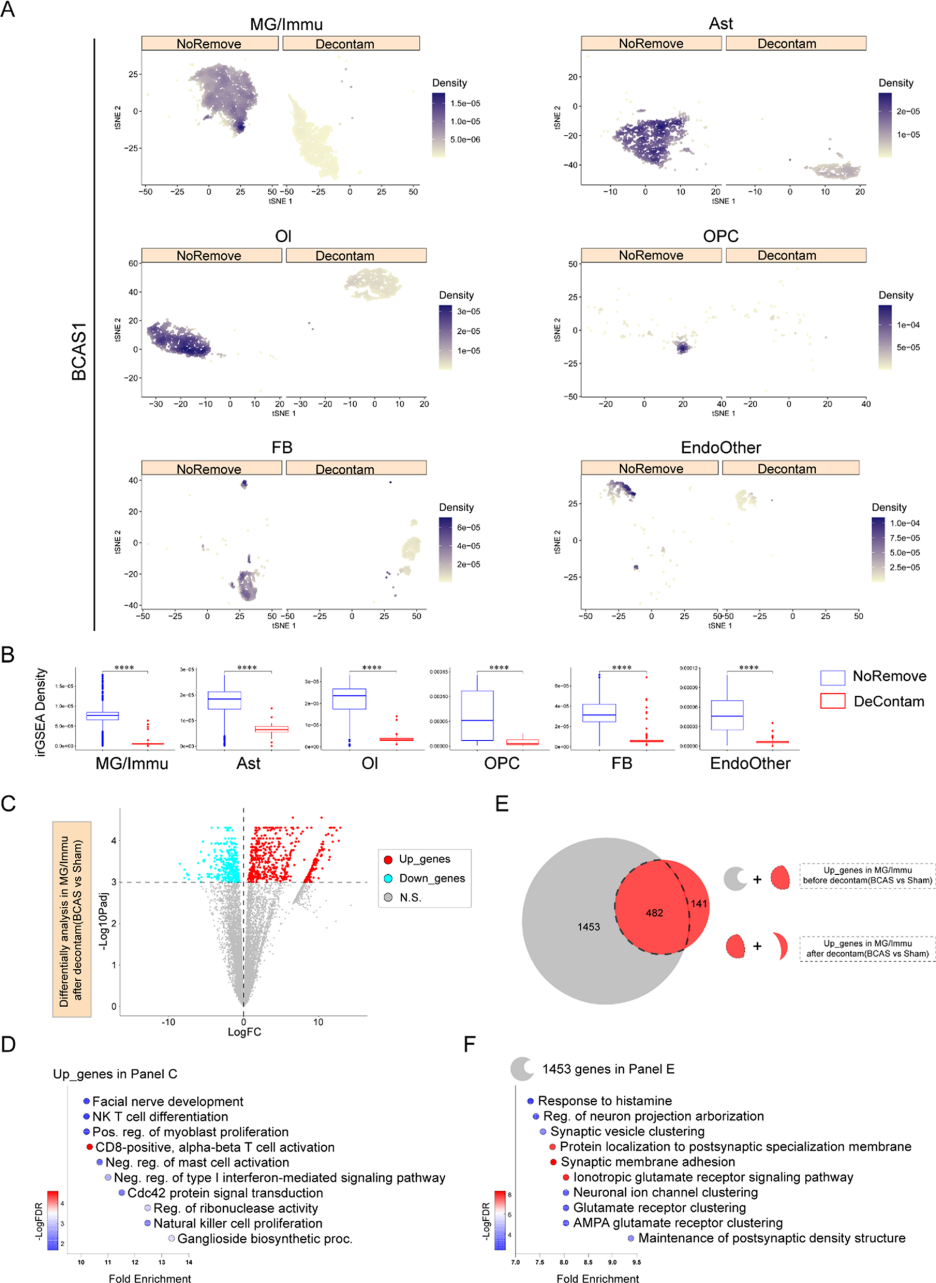
The assessment of the ambient RNAs in the non-neuronal groups before and after decontamination treatment (BCAS1). MG/Immu, microglia and other immune cell; Ast, astrocyte; Ol, oligodendrocyte; OPC, oligodendrocyte precursor cell; FB, fibroblast; EndoOther, endothelial cell and other cell. **A** t-SNE plots show the enrichment of ambient RNA markers before and after decontamination treatment in different cell types by using irGSEA analysis with the *Ucell* algorithm. **B** Box plots show the comparisons of irGSEA density of ambient RNA markers before and after decontamination in different cell types. ****P < 0.0001. **C** Volcano plot shows DEGs of MG/Immu between the sham and BCAS groups after decontamination. **D** Dot plots show the pathways enrichment of up-regulated genes of MG/Immu in the BCAS group (**C**). **E** Venn plot shows the overlap of up-regulated genes of MG/Immu in the BCAS group before and after decontamination. **F** Dot plots show the pathways enrichment of 1453 up-regulated genes only identified before decontamination, but not in after decontamination

In order to further compare the contamination degrees of non-neurons before and after decontamination, irGSEA enrichment analysis was carried out on the non-neuronal subsets of four single-nuclei libraries, and the results showed that the enrichment values of six cell subsets of the four libraries to their respective contaminated gene sets decreased significantly (Fig. 5A, B, Additional file 1: Figure S13-S15). The subsequent pseudobulk differential gene expression analysis (BCAS vs sham) was performed on decontaminated MG/Immu subsets using muscat. As shown in the volcano plot (Fig. 5C), out of the 956 differentially expressed genes, 623 were significantly up-regulated in the BCAS group compared with the sham group (Additional file 2: Table S4). Most of the up-regulated enrichment pathways in the BCAS group were related to immune response (Fig. 5D), contrasting the results before decontamination (Fig. 2J). Out of the 1935 genes up-regulated before decontamination, 482 genes remained in the up-regulated list after decontamination, while the remaining 1453 genes were eliminated. Notably, the eliminated 1453 up-regulated gene sets were mainly enriched in the neuronal functions. Furthermore, an enrichment analysis of the 141 newly discovered genes was also conducted, revealing interesting pathways such as “positive regulation of sterol transport”, “regulation of cholesterol efflux” and “positive regulation of cholesterol efflux” (Additional file 1: Figure S16 A). And, these pathways, related to “cholesterol”, were mainly contributed by the genes *Apoe*, *Zdhhc8* and *Pltp*. More importantly, as shown in the volcano plot before ambient RNAs removal, these 141 genes were masked because of their weak LogFC values (Additional file 1: Figure S16 B). However, after ambient RNAs removal, these new gene changes became significant (Additional file 1: Figure S13 C). It is speculated that these newly discovered genes, including *Apoe*, might be contributed by a small group of immune cells, which were easily masked by ambient RNAs contamination. These results indicated that the misleading results from the original contaminated microglia and other immune group analysis could be eliminated by the current analysis methods. In summary, the combination of CellBender and subcluster cleaning treatments could effectively remove the contamination of ambient RNA in non-neuronal subsets. Moreover, the in silico methods proved beneficial in exploring differentially expressed genes and related molecular pathways between the BCAS and sham groups.

### Integrative analysis of cortex-specific bulk RNA-seq and snRNA-seq revealed the important role of MG/Immu in BCAS model

**Fig. 6:**
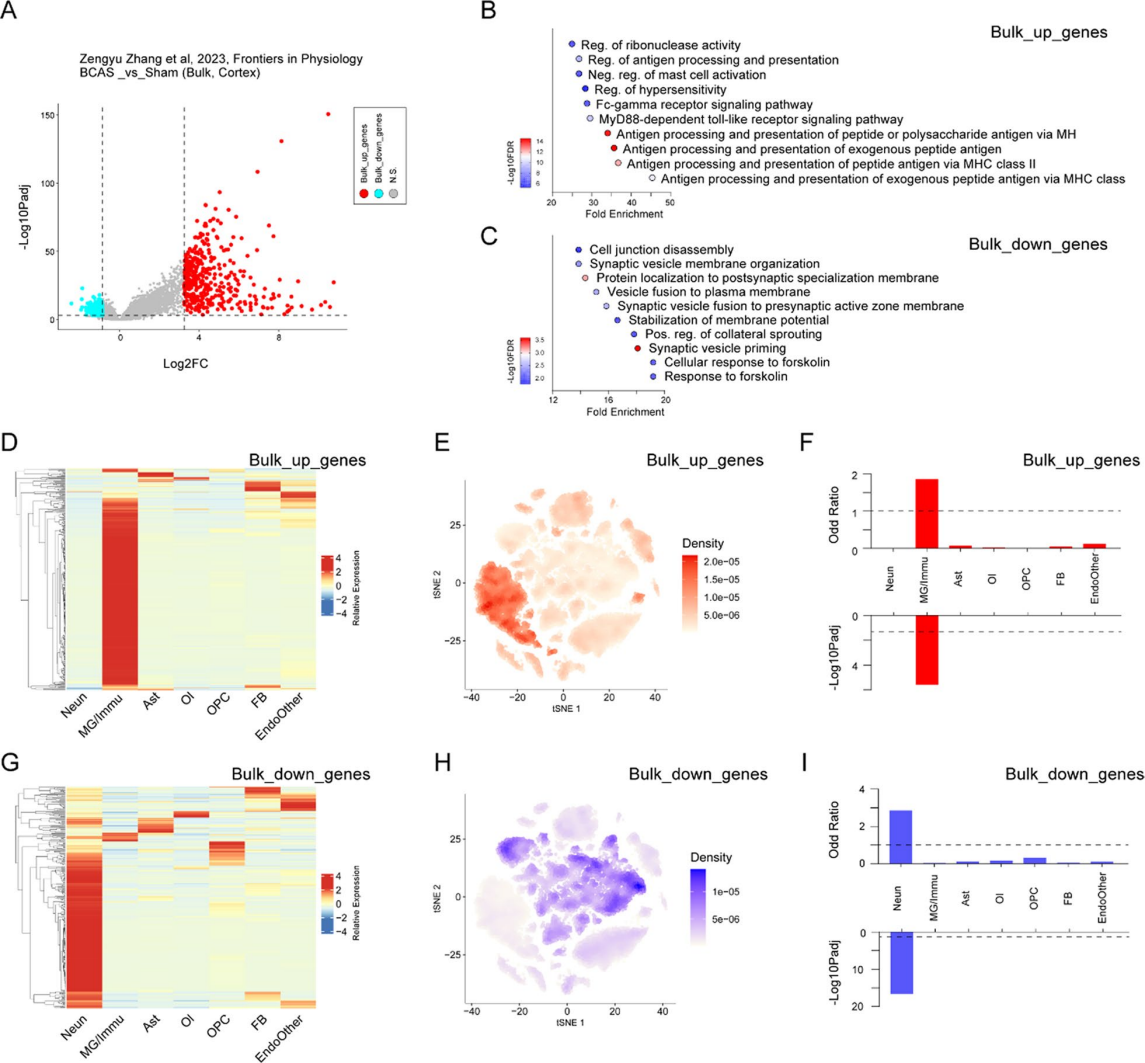
The integrative analysis of single-nuclei and bulk cortex-specific transcriptome changes after chronic deeper cerebral hypoperfusion. Neun, neuron; MG/Immu, microglia and other immune cell; Ast, astrocyte; Ol, oligodendrocyte; OPC, oligodendrocyte precursor cell; FB, fibroblast; EndoOther, endothelial cell and other cell. **A** Volcano plot shows DEGs of bulk transcriptome between the BCAS and sham groups. **B, C** Dot plots show the pathways enrichment of up- and down-regulated genes in the bulk transcriptome data (BCAS vs sham, GSE210666). **D, G** Heatmaps show snRNA-seq expression of “Bulk_up_genes” and “Bulk_down_genes” in different cell types, respectively. “Bulk_up_genes” and “Bulk_down_genes” identified in bulk transcriptome data as shown in **A**. **E, H** t-SNE plots show the enrichment of snRNA-seq for “Bulk_up_genes” and “Bulk_down_genes” by using irGSEA analysis with the *Ucell* algorithm, respectively. **F** Bar plots show the enrichment between “Bulk_up_genes” and top 200 characteristic genes in each cell type of snRNA-seq data by using GeneOverlap. **I** Bar plots show the enrichment between “Bulk_down_genes” and top 200 characteristic genes in each cell type of snRNA-seq data by using GeneOverlap

According to our previous bulk transcriptome data of BCAS model, neuroinflammation played an important role in the process of chronic hypoperfusion caused by carotid artery stenosis [19, 20]. In fact, microglia, astrocytes and other cells can play an important role in neuroinflammation. However, due to the limitations of bulk RNA-seq, our previous study did not describe the main effector cells during the neuroinflammation of chronic hypoperfusion. To address this, we integrated the cortex-specific snRNA-seq in this study and our previous bulk RNA-seq to identify the key cell types involved in important pathways. After downloading the GEO database (GSE210666), we performed the DEGs analysis of cortex-specific bulk RNA-seq between the BCAS and sham groups. The top 2.5% up- and down-regulated genes were selected as DEGs, referred to as “Bulk_up_genes” and “Bulk_down_genes”, respectively. Out of the 696 DEGs, 371 were significantly up-regulated and 325 were significantly down-regulated (Fig. 6A, Additional file 2: Table S5). Among them, the gene set “Bulk_up_genes” were mainly involved in antigen presentation and other related immune pathways, while the gene set "Bulk_down_genes" was associated with neuronal functions (Fig. 6B, C). According to the heatmaps (Fig. 6D, G), we found that most genes in the “Bulk_up_genes” set were highly expressed in the MG/Immu group of snRNA-seq from this study, whereas most genes in the “Bulk_down_genes” set were most expressed in the neuron group of snRNA-seq. Additionally, irGSEA enrichment analysis also showed that MG/Immu subsets were mainly enriched in “Bulk_up_genes”, and that neuronal subsets were mainly enriched in “Bulk_down_genes” (Fig. 6E, H). Furthermore, by using the GeneOverlap package, we performed enrichment analysis of “Bulk_up_genes” and “Bulk_down_genes: for the characteristic gene sets of each group of snRNA-seq. The analysis revealed that the set of “Bulk_up_genes” was mainly enriched in MG/Immu group (odds ratio: 1.86; -Log10Padj: 5.60), and that the set of “Bulk_down_genes” was mianly enriched in neuronal group (odds ratio: 2.86; -Log10Padj: 16.59) (Fig. 6E, H). These results suggested that MG/Immu played an important role in the chronic neuroinflammatory reaction induced by severe carotid artery stenosis, as revealed in the cortex-specific bulk RNA-seq data. In addition, given that the increase in the number of MG/Immu subsets was the largest in this disease state (Fig. 4B), it was recommended to focus subsequent analyses on the MG/Immu groups.

### The subgroup analysis of MG/Immu group revealed *Apoe*^+^ microglia/macrophage subgroup mediated by severe carotid artery stenosis

**Fig. 7:**
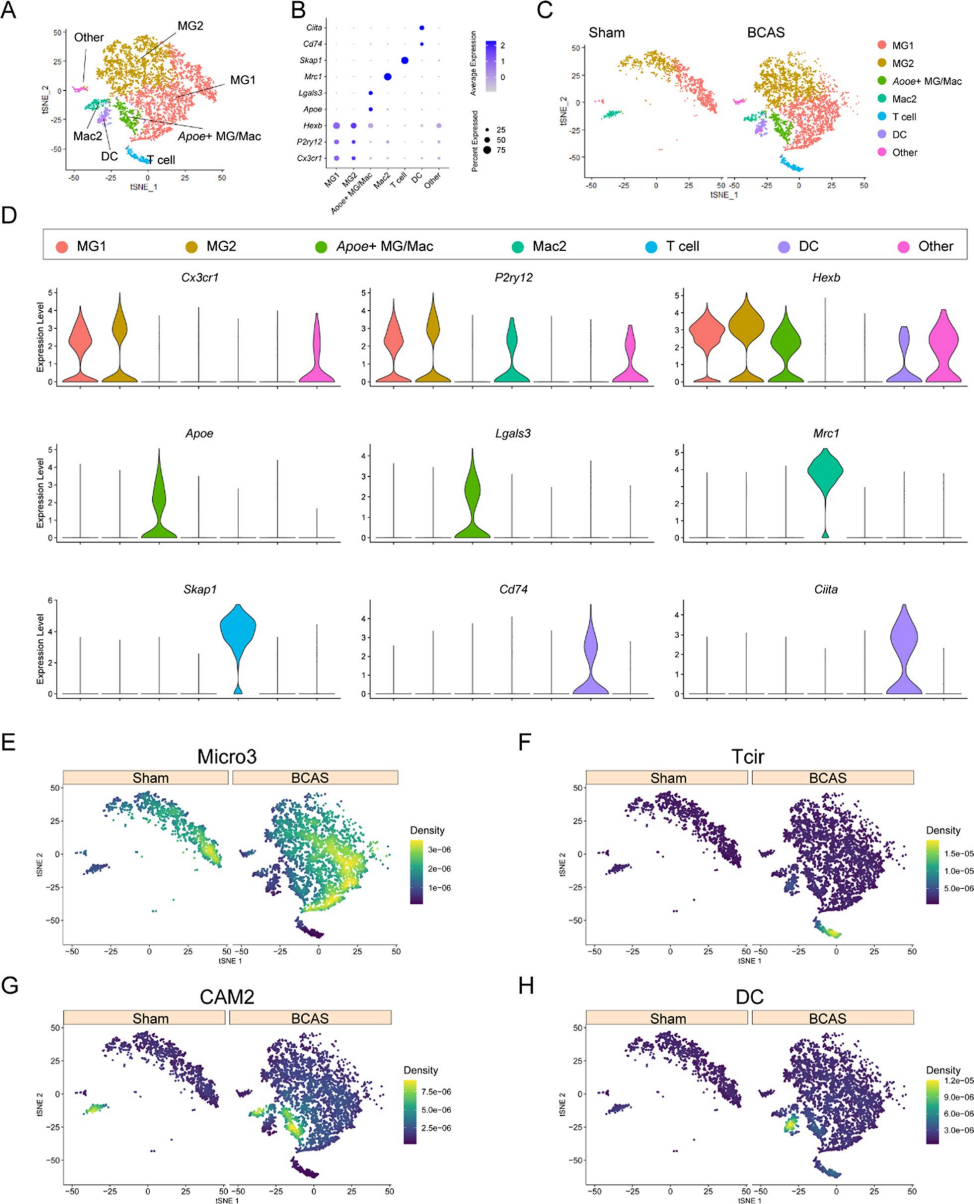
Subgroup analysis of MG/Immu of sham/BCAS mice at single-nuclei resolution. In our study, MG1, microglia 1; MG2, microglia 2; *Apoe*^+^ MG/Mac: *Apoe*^+^ microglia/macrophage; Mac2: macrophage; DC, dendritic cell. In published data (GSE189432), Micro3, microglia 3; Tcir, circulating T cells; CAM2, central nervous system-associated macrophages2; DC, dendritic cell. **A** t-SNE plots show all single-nuclei of MG/Immu in all samples. **B** Dot plots show the average expression of specific genes in different groups. **C** t-SNE plots show all single-nuclei of MG/Immu in the sham and BCAS groups, respectively. **D** Violin plots show the expression of *Syt1* and *Grin2b* in different subgroups of MG/Immu. **E** t-SNE plots show the enrichment of MG/Immu subgroup in our snRNA-seq data for marker genes of Micro3 (GSE189432) by using irGSEA analysis with the *AUcell* algorithm. **F** t-SNE plots show the enrichment of MG/Immu subgroup in our snRNA-seq data for marker genes of Tcir (GSE189432) by using irGSEA analysis with the *AUcell* algorithm. **G** t-SNE plots show the enrichment of MG/Immu subgroup in our snRNA-seq data for marker genes of CAM2 (GSE189432) by using irGSEA analysis with the *AUcell* algorithm. **H** t-SNE plots show the enrichment of MG/Immu subgroup in our snRNA-seq data for marker genes of DC (GSE189432) by using irGSEA analysis with the *AUcell* algorithm

Based on the above findings, microglia and other immune cells played an important role in severe carotid artery stenosis, so we isolated MG/Immu subsets for the further analysis. Further subgroup analysis revealed the presence of additional immune populations within the MG/Immu group (Fig. 7). As shown in the t-SNE plot (Fig. 7A), the MG/Immu group could be further divided into seven cell subsets, namely MG1, MG2, *Apoe*^+^ MG/Mac, Mac2, T cells, dendritic cells (DCs), and other cell subsets. Moreover, the marker genes such as *Cx3cr1*, *P2ry12*, *Hexb*, *Apoe*, *Lgals3*, *Mrc1*, *Skap1*, *Cd74* and *Ciita* were highly expressed in their respective nuclei subsets (Fig. 7B and D). Furthermore, the number of nuclei in the MG1 and MG2 populations in the BCAS group increased when compared with the sham group, while DCs, T cells and *Apoe*^+^ MG/Mac subsets were only present in the BCAS group, but not in sham group (Fig. 7C). To improve the accuracy of inflammatory cell annotation, we used the scRNA-seq database (GSE189432) of MCAO model in the published article for further joint analysis. We found that MG1 and MG2 groups in this project were enriched in Micro3 groups in the MCAO model (Fig. 7E). The T cell subsets of this project were enriched in the Tcir group in the MCAO model (Fig. 7F). Two subsets of Mac2 and *Apoe*^+^ MG/Mac in this project were enriched in CAM2 subset in the MCAO model (Fig. 7G). The DC subsets in this project are enriched in the DC subsets in the MCAO model (Fig. 7H). At the same time, we carried out the related pathway enrichment analysis on the characteristic gene sets of their respective subpopulations (Additional file 1: Figure S17).

**Fig. 8:**
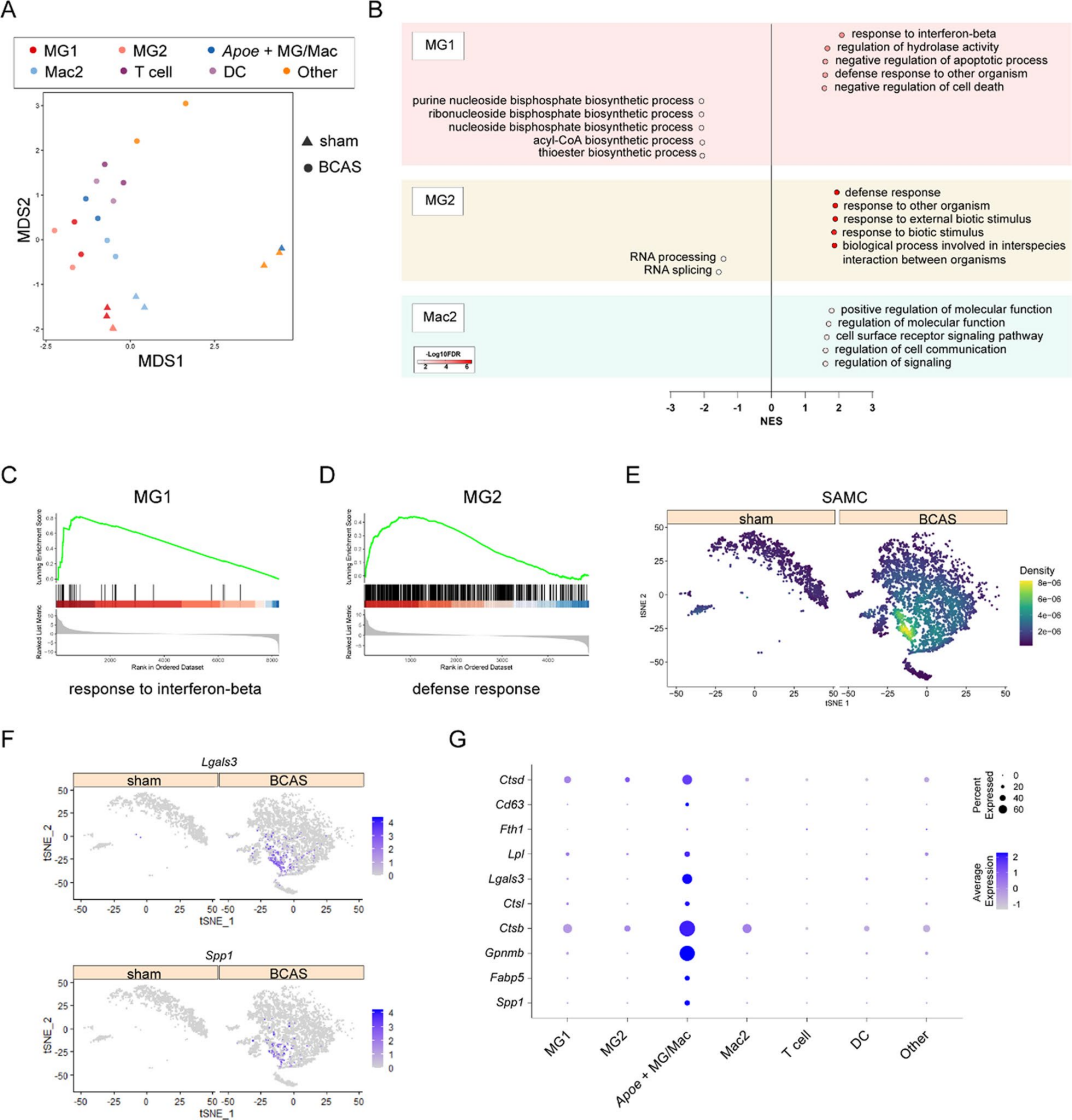
Differentially genes expressed analysis of MG/Immu between the BCAS and sham groups. **A** MDS (Multidimensional Scaling) plots show the separation MG/Immu subgroups between the BCAS and sham groups. MG1, microglia 1; MG2, microglia 2; *Apoe*^+^ MG/Mac: *Apoe*^+^ microglia/macrophage; Mac2: macrophage; DC, dendritic cell; Other, other cells. **B** Dot plots show the gene set enrichment analysis (GSEA) of up- and down- regulated functional pathways of three microglia subsets in the BCAS group. **C** GSEA plot shows the positive enrichment of the “response to interferon-β” pathway of MG2 in the BCAS groups. **D** GSEA plot shows the positive enrichment of the “defense response” pathway of MG1 in the BCAS groups. **E** t-SNE plots show the enrichment of MG/Immu subgroup in our snRNA-seq data for marker genes of SAMC (GSE189432) by using irGSEA analysis with the *AUcell* algorithm. **F** t-SNE plots show the expression of *Lgals3* and *Spp1* in different MG/Immu subsets between the sham and BCAS groups. **G** Dot plots show the average expression specific genes in different MG/Immu subsets

Subsequently, we used muscat to analyze the differential genes of pseudobulk in different subsets of MG/Immu. Multidimensional scaling (MDS) analysis showed that different MG/Immu subsets were divided into two subsets based on the treatments of sham and BCAS (Fig. 8A). Since there were few or no nuclei of some subsets in sham group, we only carried out pseudobulk differential gene analysis of MG1, MG2 and Mac2. The results of pathway enrichment analysis suggested that the up-regulated genes in the BCAS group were significantly enriched in “response to interferon-β” pathway compared to the sham group. This was consistent with our previous published bulk RNA-seq data [19, 20], suggesting that the previously found interferon-related pathways were mainly mediated by the MG1 subset (Fig. 8B, C). And, MG2 microglia subsets were involved in immune response, exogenous stimulation and other related pathways (Fig. 8B, D).

Among the three types of immune nuclei subsets that only appeared in the BCAS group, one group of nuclei exhibited high expression of *Apoe* gene at the transcriptional level (Fig. 7A, B), and participated in lipid metabolism-related signaling pathways such as “low density lipoprotein particle remodeling” (Additional file 1: Figure S17C). Previous studies have reported that a group of cell subsets named stroke-associated myeloid cells (SAMCs) were found in MCAO model, which were partly derived from intracranial microglia and partly from peripheral immune infiltration [27]. At the same time, SAMCs are primarily involved in lipid metabolism pathways, and their core genes play important roles, including *Spp1*, *Fabp5*, *Gpnmb*, *Ctsb*, *Ctsl*, *Lgals3*, *Lpl*, *Fth1*, *Cd63* and *Ctsd* [27]. Interestingly, we carried out irGSEA enrichment analysis of single-nuclei data of MG/Immu group for the top 200 highly expressed marker genes of SAMCs in MCAO model, and found that *Apoe*^+^ MG/Mac in this study showed the highest enrichment in SAMCs marker genes (Fig. 8E). Moreover, compared to other subsets, *Apoe*^+^ MG/Mac exhibited the highest expression of the aforementioned core genes (Fig. 8F, G). These results suggested that we could also identify a group of cell subsets with similar functions to those in MCAO model by using the snRNA-seq technique.

Meanwhile, in order to compare the difference of MG/Immu subgroup analysis between before and after ambient RNAs removal, we also conducted a similar subgroup analysis in the initial MG/Immu population without ambient RNA removal. As shown in the t-SNE plots (Additional file 1: Figure S18 A), without ambient RNAs removal, the MG/Immu group could be further divided into 11 subgroups (MG/Immu0, MG/Immu1, MG/Immu2, MG/Immu3, MG/Immu4, MG/Immu5, MG/Immu6, MG/Immu7, MG/Immu8, MG/Immu9 and MG/Immu10). Unlike the subgroup analysis after ambient RNAs removal (Fig. 7A–C), there was little overlap of the sham and BCAS groups in any of the MG/Immu subgroups (Additional file 1: Figure S18 A). Among these 11 subgroups, the MG/Immu3, MG/Immu8 and MG/Immu10 subgroups mainly consisted of the droplets containing ambient RNAs (Additional file 1: Figure S18 B, D), and were enriched in neuronal related pathways (Additional file 1: Figure S19). The subgroups MG/Immu0, MG/Immu1, MG/Immu2 and MG/Immu8 were composed of MG1 and/or MG2, accompanied with some ambient RNAs (Additional file 1: Figure S18 C, D), and the enrichment analysis of characteristic genes of these subgroups were also shown (Additional file 1: Figure S20). Notably, the subgroup MG/Immu0 was enriched in the neuronal related pathways (Additional file 1: Figure S20 A), suggesting that many nuclei in MG1 and MG2 were masked by ambient RNAs. According to the dot plots (Additional file 1: Figure S18 E), the expression of ambient genes was lower in the MG/Immu2 subgroup, which mainly consisted of the nuclei in the sham group. We speculated that the total separation between the sham and BCAS groups in the t-SNE plots may also be attributed to the different contamination conditions between the two conditions. Thus, we extracted the subgroups MG/Immu0 and MG/Immu2, and performed DEGs analysis between the BCAS and sham groups. As expected, the up-regulated genes in the BCAS group also participated in neuronal related pathways (Additional file 1: Figure S21 A). And, similar results were observed when analyzing the subgroups MG/Immu1 and MG/Immu2 (Additional file 1: Figure S21 B).

In the remaining subgroups, the MG/Immu4 mainly corresponded to the *Apoe*^+^ MG/Mac group in the DeContam object, the MG/Immu5 corresponded to the T cell group, the MG/Immu6 corresponded to the DC group, the MG/Immu9 corresponded to the Mac2 group (Additional file 1: Figure S18 C, D). The corresponding subgroups before and after ambient RNAs removal showed the similar biological pathways (Additional file 1: Figure S17 C-F, Additional file 1: Figure S22 A-D), which were contributed by the shared gene sets between the two corresponding subgroups (Additional file 1: Figure S22 E). However, compared to the subgroup before ambient RNAs removal, the LogFC values of shared characteristic genes significantly increased (Additional file 1: Figure S22 F). For example, among the characteristic genes of MG/Immu4 in the NoRemove object, the gene *Apoe* ranked at the bottom position. However, among the characteristic genes of *Apoe*^+^ MG/Mac in the DeContam object (Additional file 1: Figure S23 A), the gene *Apoe* ranked at the topper position (Additional file 1: Figure S23 B), which was in line with our previous findings (Additional file 1: Figure S16).

### The biological function of *Apoe*^+^ MG/Mac subpopulation was further analyzed by pseudo-time analysis

**Fig. 9:**
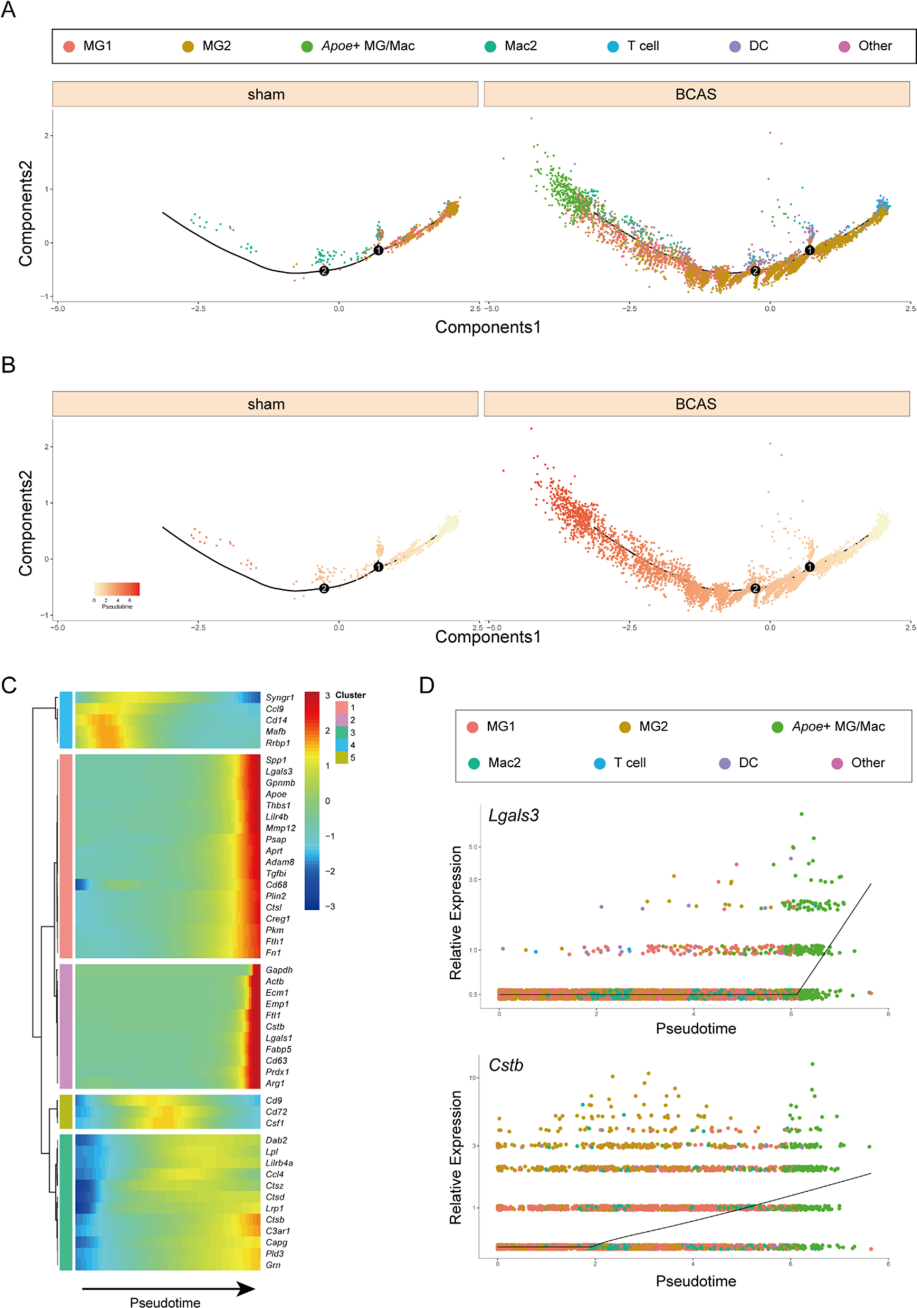
Pseudo-time analysis of different MG/Immu subsets by Monocle 2 algorithm. MG1, microglia 1; MG2, microglia 2; *Apoe*^+^ MG/Mac: *Apoe*^+^ microglia/macrophage; Mac2: macrophage; DC, dendritic cell; Other, other cells. **A** Distribution of MG/Immu in 7 different subsets. **B** Distribution of MG/Immu subsets according to pseudo-time value. **C** Heatmaps show genes of 50 marker genes of SAMC (GSE189432) that dynamically change with pseudo-time value. **D** Genes (*Lgal3* and *Cstb*) that dynamically changes with pseudo-time value

To investigate the evolution of *Apoe*^+^ MG/Mac subpopulation, we selected top 200 characteristic genes from SAMC in MCAO model as the core gene set for pseudo-time analysis. As shown in Fig. 9A, B, MG2 gradually transitioned into MG1 subtype over pseudo-time, and subsequently, MG1 gradually evolved into *Apoe*^+^ MG/Mac subgroup. Considering the roles of MG1, MG2 and *Apoe*^+^ MG/Mac subsets in chronic hypoperfusion (Figs. 8A, B, 9B–D), we speculated that chronic cerebral hypoperfusion might activate the immune response function of microglia, then leading to the upregulation of the interferon-related pathway involved in microglia. Eventually, the activated microglia, accompanied with macrophages, participated in lipid metabolism pathway, potentially helping microglia/macrophage engulf lipid-rich debris. Among them, we selected top 50 characteristic genes in SAMCs for visualize the changes of gene expression over pseudo-time. As shown in the heatmap (Fig. 9C), except for cluster4, which consisted of only 5 genes, the genes in the cluster 3 and 5 (including about 1/3 genes) gradually reach the peak of expression with pseudo-time. Besides, most genes (in the cluster1 and 2) are suddenly up-regulated at the late stage of pseudo-time series, including genes such as *Lgals3* and *Cstb* (Fig. 9C, D).

## Discussion

In our current study, we chose bilateral carotid artery stenosis mice as our models to assess the ambient RNAs contamination of snRNA-seq under diseased conditions. If the ambient RNAs contamination was not effectively removed, it would largely contribute to improper cell annotation and the following DEGs analysis. Notably, the ambient RNAs contamination could be greatly minimized, by using the combination of CellBender and additional subcluster cleaning. Thus, the transcriptome of chronic cerebral hypoperfusion caused by severe carotid artery stenosis was well constructed at single-nuclei resolution in our study. This approach enabled us to identify cell-type-specific transcriptional changes and gain insights into neuroinflammation associated with chronic cerebral hypoperfusion.

It is important to consider the advantages and disadvantages of scRNA-seq and snRNA-seq, respectively. In the process of scRNA-seq library construction of CNS, neurons are more vulnerable than other cell types, and they can be damaged easily when preparing the single cells/nuclei suspension. Even if they are not damaged, the neurons are irregularly shaped and relatively large, which cannot be captured effectively by the 10X genomics instrument, thus resulting in the bias of cell components. Moreover, in the recent years, accumulating studies have shown that in the process of cell dissociation from brain tissue to single cell suspension, enzymatic hydrolysis reagents can easily induce the production of ex vivo “activated” microglia (exAM), which does not exist in organisms, and the transcriptional activation of stress-related genes [29]. Although snRNA-seq technology cannot effectively capture the information of cytoplasmic mRNA, yet if the intron sequence analysis is included during the analysis of snRNA-seq data, it will achieve equivalent gene detection when compared with scRNA-seq analysis for the further analysis of biological functions [30]. However, Nicola Thrupp et al. [31] also held the opposite view that snRNA-seq is not suitable for the investigation of microglia activation. According to their results, although little difference was found in abundance between microglial nuclei and whole cells, a small but important group of genes was depleted in nuclei, such as *APOE*. In fact, they also found that nuclei-abundant genes, such as *RBFOX1*, were enriched in neuronal and synaptic terms, suggesting synaptosome contamination. In contrast with the view of Nicola Thrupp et al., Gerrits et al. [32] demonstrated that the similar profiles were found between microglial whole cells and nuclei, after the FACS technique was employed. However, in the study of Nicola Thrupp et al., the missing of some important genes might be also masked by synaptosome contamination [31], and physical isolation is also a good tool to help remove ambient RNA and detect some cell types masked by ambient RNAs according to the results of Gerrits et al. [9]. More importantly, according to our results, *Apoe* and other important activation genes of the MG/Immu subgroup could be also identified after in silico decontamination. Thus, when combined with physical sorting or the in silico method to remove ambient RNA contamination, snRNA-seq becomes a valuable alternative for investigating neuroinflammation.

In our research, in order to prevent the degradation of nucleus RNA, it took only about 30 min from the brain tissue dissection to the preparation of single-nuclei suspension, and RNase inhibitor was also added to the tissue lysate. According the peak maps of cDNA and library (Additional file 1: Figure S5-S7, Additional file 2: Table S2), all samples passed the quality controls of 10X genomics, suggesting that the RNA of the four samples was not degraded, and that library ligation products were correct. As expected, the BCAS group samples exhibited more pronounced ambient RNAs contamination than the sham group samples, with the ambient RNA primarily originating from neuronal transcripts (Figs. 2K, 5F). These results suggested that in BCAS group, many neurons were undergoing cell death, leading to pathological nuclei breakage. Therefore, we found the misleading results that the up-regulated genes in the MG/Immu group were enriched in neuronal functions after performing DEGs analysis (BCAS vs sham) without ambient RNA removal. Walter Muskovic et al. [33] found that dead cells or empty droplets with only ambient RNA contamination contain low UMI number. This result was similar to the droplets removed by CellBender software (Fig. 3E), suggesting that the CellBender software could remove low-quality droplets populations and avoid misleading subsequent analysis. Notably, in the context of cerebral ischemia, astrocytes can express markers of neural stem cells, making it challenging to determine whether they have biological functions under ischemic conditions or are indeed contaminated by ambient RNA released from damaged neurons. Since microglia is a class of non-nervous system derived cell, even in the case of cerebral ischemia, it mainly shows strong immune response, but few neuron-related transcriptional features. Therefore, microglia in brain tissue under ischemic conditions serve as a good model to evaluate the efficiency of removing ambient RNAs. Interestingly, this in silico method could greatly reduce the ambient RNAs in MG/Immu population under ischemia challenge. Moreover, the DEGs analysis of MG/Immu groups revealed that many nervous system-related pathways were enriched in BCAS group before decontamination (Fig. 2L), while the removal of ambient RNA could largely eliminate this misleading result (Fig. 5E). Thus, the in silico method of CellBender and subsequential subcluster cleaning can not only remove the ambient RNA of snRNA-seq in non-diseased state as previously reported [9], but also tackle ambient RNA contamination in single-nuclei datasets from damaged brain tissues.

The comprehensive analysis of the source of ambient RNAs in the BCAS group also shed light on the related pathophysiology of chronic cerebral hypoperfusion. In conditions of brain injury, microglia are primarily responsible for clearing cell debris from damaged neurons [34, 35]. Once the accumulation rate of neuronal debris exceeds the microglial clearance rate, neurological deficits will occur [36]. Meanwhile, the removal of microglial debris has also gradually gained attention in the recent years. In normal neurophysiological states of mouse and human, the microglial turnover rate is approximate 30% in each year [37, 38]. Under the ischemic stroke conditions, the number of microglia decreases rapidly via the apoptotic process after the sustained parenchymal microglia proliferation at the early stage [39]. Furthermore, even if numerous microglia are depleted in a short time by PLX5622 administration, there is insufficient direct evidence that microglial corpses accumulate in the CNS. Interestingly, as shown in a recent study, microglial debris can be removed by astrocytes via C4b-facilitated phagocytosis, followed by the degradation RUBICON-dependent noncanonical autophagy [40]. Unlike the clearance of neuronal debris, the removal of microglial debris by astrocytes is governed by a more efficient machinery in order to maintain the CNS homeostasis. Thus, we speculated that there existed more neuronal corpses than microglial corpses in the BCAS group. And as a result, ambient RNAs contamination predominantly consisted of neuronal transcripts, accompanied with some microglial transcripts.

Our previous studies, along with others’, have consistently reported that the cerebral blood flow in severe bilateral carotid artery stenosis mouse model was seriously reduced [19–22]. In addition, the BCAS mouse model showed serious spatial learning and memory damage, accompanied with various pathological phenotypes such as neuronal loss, activation of astrocytes and microglia, and infiltration of other immune cells. Cortex-specific bulk RNA-seq also suggested that severe carotid artery stenosis can cause strong immune response transcriptomic maps, but the cell types involved in it are not completely clear [19, 20]. According to the integrative analysis of our previous bulk RNA-seq data and snRNA-seq in this study, it was found that the up-regulated gene set caused by severe carotid artery stenosis in bulk RNA-seq was mainly enriched in the group of microglia and other immune cells, suggesting that they mainly participated in the immune response in this pathological state. Our previous studies [19] have found that interferon pathway was significantly up-regulated in the cortex of severe carotid artery model, and this study further showed that the microglia subtype involved in this pathway was MG1 group. Moreover, other microglia and immune subsets also participated in other different biological functions in severe chronic cerebral hypoperfusion.

More importantly, because of the limitations of bulk RNA-seq techniques, we might miss subtle gene changes induced by some small subgroups that were also important for chronic cerebral ischemia-induced brain functional abnormality. Interestingly, by using the snRNA-seq techniques, we also found a novel subgroup of *Apoe*^+^ MG/Mac, and their transcriptomic features were similar to the transcription map of SAMCs found in MCAO. This cell subset mainly manifested the molecular phenotypes related to lipid metabolism and phagocytosis, suggesting that it might be mainly responsible for clearing lipid-enriched debris. Studies have shown that many transcriptional features of SAMCs are conserved in many species including humans [27, 41, 42]. Notably, it was found that the SAMCs occur in the short time window (within 72 h) of ischemia time in MCAO model [27]. However, in the severe carotid artery stenosis model of this study, cerebral blood flow hypoperfusion lasted for 3 weeks, which belongs to the pathological process of chronic cerebral ischemia, and similar cell groups were found. Based on the evidence of previous studies and this study, the *Apoe*^+^ MG/Mac cell subsets were conservatively present in different species, different cerebral ischemia models and different cerebral ischemia time, suggesting that the *Apoe*^+^ MG/Mac subgroup play an important role in the process of cerebral ischemia.

In fact, many studies on neurodegenerative diseases, including Alzheimer's disease, have shown that in both clinical and related animal models the gene *Apoe* can affect the phenotype related to lipid metabolism of microglia and then participate in the process of neuropathological phenotype [43, 44]. The main apolipoprotein synthesized in the CNS, ApoE plays a neuroprotective role in the conditions of experimental subarachnoid hemorrhage by participating in reducing early brain injury via microglial quiescence [45]. In addition, ApoE-mimetic peptide can also serve as the ligand of triggering receptor expressed on myeloid cells 2 (TREM2), and as a result, the activation of TREM2 can improve neurological functions and attenuate neuroinflammation as well as neuronal apoptosis in the mouse model of intracerebral hemorrhage [46]. In contrast to these two studies on cerebral hemorrhage [45, 46], Carolin Beuker [27] found that the microglia/macrophages participating in lipid metabolism played an harmful role in ischemic stroke animal models, as evidence that blocking markers specific for this group of cells attenuated brain injury. Despite of the controversial roles of *Apoe* in brain injuries among different studies, all studies suggested consistently that *Apoe* and other genes related to lipid metabolism can influence microglial phagocytosis. That is to say, whether *Apoe* plays a benefit or harmful role in brain injuries depends on the role of microglial phagocytosis at certain disease conditions. On one hand, the engulfment function of microglia can clean dead neurons, and cell/myelin debris to exert neuroprotective effect against brain damages; on the other, this phagocytosis function can also break down blood brain barrier and swallow viable neurons [47].

In fact, there are some strengths of this combined in silico approach. This combined cleaning technique can avoid misleading clustering, and identify some underestimated genes. Specifically, in other ischemic stroke models, scRNA-seq data in the subgroup of microglia show that there still exist some subgroups composed of the nuclei from normal and diseased conditions [48, 49]. According to our previous neuropathology investigations [19], although dramatic microgliosis could be found in the BCAS model, yet there still existed non-activated microglia. Thus, the total separation of MG/Immu in the NoRemove object might result from ambient RNAs contamination, but not the diseased conditions. However, the issues of misleading clustering and some population masked by ambient RNAs could be reduced to a large extent by using the in silico approach (Fig. 7A, C). In addition, although some small subgroups could be also found without ambient RNAs removal, yet some important characteristic genes of these subgroups would be masked if ambient RNAs were not cleaned. However, it is important to acknowledge the limitation of our study, namely the small sample size (*n* = 2 libraries/condition). In order to improve the representativeness of each snRNA-seq library, each biological replicate was from two mice, so four mice were used in either Sham or BCAS group. Our single-nuclei suspension preparation yielded similar concentrations (Sham1: 1250/μl, Sham2: 1070/μl, BCAS1: 1070/μl, BCAS2: 1090/μl), and low and similar clump rates across all samples (sham1: 2.21%, sham2: 2.38%, BCAS1: 2.7%, BCAS2: 1.99%) (Additional file 2: Table S1). Considering the capture efficiency of 10X Genomics system, we uploaded 20,000 nuclei of each sample, and expected that the final average number of captured nuclei of each sample was more than 10,000 (Additional file 2: Table S1). Although some nuclei might be classified as contaminated by mistake and depleted, yet the final number of each group was enough for the further analysis after ambient RNAs removal (sham: 21,934, BCAS: 20,235, total: 42,169). The quality control data provided reassurance regarding the methodological stability of our snRNA-seq library construction. By using the *Seurat*’s function, *FindAllMarkers*, nuclei were treated as samples in order to identify the characteristic gene sets of each group/subgroup. For example, since the subgroup *Apoe*^+^ microglia/macrophage only existed in the BCAS group, *Apoe*^+^ microglia/macrophages (*n* = 488) were compared with other microglia/immune cells (*n* = 6840) to identify the core molecular function of this subgroup (Fig. 7A). In this situation, the sample size of each group was not small. However, the statistical power and accuracy of the results were compromised in the pseudobulk differential expression analysis, where each snRNA-seq library was treated as a single sample. Our current study only increased researchers’ awareness of the importance of ambient RNAs removal when performing snRNA-seq analysis, especially in diseased conditions. Thus, future studies with a larger sample size are warranted to enhance statistical power and result accuracy.

In summary, our study provides with a comprehensive analysis of ambient RNAs contamination in diseased conditions, found that the in silico approaches are sufficient to effectively remove the contamination. The results shed light on the distinct roles played by different microglia and immune cell subsets in the immune response and pathological processes associated with chronic cerebral hypoperfusion. The discovery of the *Apoe*^+^ microglia/macrophage subgroup highlights the potential involvement of lipid metabolism and phagocytosis in the pathogenesis of cerebral ischemia. However, further investigations are needed to fully elucidate the functional significance of these cell subsets and their potential as therapeutic targets for cerebral ischemia-related disorders. The ambient RNAs removal is critical for accurate analysis of the downstream analysis, and is worthy of widespread applications in other single-nuclei studies on neuropsychiatric and neurodegenerative disorders.

## Supplementary Information

**Additional file 1: Figure S1.** Microscopic examinations of the nuclei suspension of the sham1 sample. A Microscopic image in different channels. FL1 showing live cells, FL2 showing dead cells. B Bar plots showing the number of nuclei in different sizes according to the images of bright field. C, D Bar plots showing the number of nuclei in different RFUs of FL1and FL2, respectively. RFU, relative fluorescence units. **Figure S2.** Microscopic examinations of the nuclei suspension of the BCAS1 sample. A Microscopic image in different channels. FL1 showing live cells, FL2 showing dead cells. B Bar plots showing the number of nuclei in different sizes according to the images of bright field. C, D Bar plots showing the number of nuclei in different RFUs of FL1and FL2, respectively. RFU, relative fluorescence units. **Figure S3.** Microscopic examinations of the nuclei suspension of the sham2 sample. A Microscopic image in different channels. FL1 showing live cells, FL2 showing dead cells. B Bar plots showing the number of nuclei in different sizes according to the images of bright field. C, D Bar plots showing the number of nuclei in different RFUs of FL1and FL2, respectively. RFU, relative fluorescence units. **Figure S4.** Microscopic examinations of the nuclei suspension of the BCAS2 sample. A Microscopic image in different channels. FL1 showing live cells, FL2 showing dead cells. B Bar plots showing the number of nuclei in different sizes according to the images of bright field. C, D Bar plots showing the number of nuclei in different RFUs of FL1and FL2, respectively. RFU, relative fluorescence units. **Figure S5.** Gel electrophoresis of different samples in different lanes as measured by an Agilent 4200. C1, the cDNA products of sham1; C2, the cDNA products of BCAS1; C3, the cDNA products of sham2; C4, the cDNA products of BCAS2; C5, the snRNA-seq library of sham1; C6, the snRNA-seq library of BCAS1; C7, the snRNA-seq library of sham2; C8, the snRNA-seq library of BCAS2. **Figure S6.** The reverse transcription products fragment sizes of different samples as measured by an Agilent 4200. **Figure S7.** The snRNA-seq libraries fragment sizes of different samples as measured by an Agilent 4200. **Figure S8.** The barcode rank plots of four different samples generated by the software CellRanger. **Figure S9.** The expression of microglia-specific gene in the sham and BCAS group.** A** t-SNE plots show the expression of *Hexb* in different groups between the sham and BCAS groups. **Figure S10.** The analysis of the features of the droplets depleted from MG/Immu in the BCAS group. A, B T-SNE plot shows single-nuclei in all droplets in the MG/Immu of the BCAS group before ambient RNAs removal. C Volcano plot shows DEGs of MG/Immu between the depleted and retained droplets. D, E Dot plots show the pathways enrichment of the genes enriched in depleted and retained droplets, respectively. **Figure S11.** The analysis of the features of the droplets depleted from MG/Immu in the BCAS group. A Volcano plot shows DEGs of the droplets depleted by CellBender between the BCAS and sham groups. B Dot plots show the pathways enrichment of up-genes enriched in panel A. C Dot plots show the average expression of specific genes in the droplets depleted by CellBender between the BCAS and sham groups. **Figure S12.** Pathways enrichment analysis of different cell types. Neun, neuron; MG/Immu, microglia and other immune cell; Ast, astrocyte; Ol, oligodendrocyte; OPC, oligodendrocyte precursor cell; FB, fibroblast; EndoOther, endothelial cell and other cell. A-G Dot plots show the pathways enrichment of top 200 characteristic genes in different cell types. H Heatmap shows the expression of top 200 characteristic genes in different cell types. **Figure S13.** The assessment of the ambient RNAs of the sham1 sample in the non-neuronal groups before and after decontamination treatment. MG/Immu, microglia and other immune cell; Ast, astrocyte; Ol, oligodendrocyte; OPC, oligodendrocyte precursor cell; FB, fibroblast; EndoOther, endothelial cell and other cell. A t-SNE plots show the enrichment of ambient RNA markers before and after decontamination treatment in different cell types by using irGSEA analysis with the *Ucell* algorithm. B Box plots show the comparisons of irGSEA density of ambient RNA markers before and after decontamination in different cell types. ****P < 0.0001. **Figure S14.** The assessment of the ambient RNAs of the sham2 sample in the non-neuronal groups before and after decontamination treatment. MG/Immu, microglia and other immune cell; Ast, astrocyte; Ol, oligodendrocyte; OPC, oligodendrocyte precursor cell; FB, fibroblast; EndoOther, endothelial cell and other cell. A t-SNE plots show the enrichment of ambient RNA markers before and after decontamination treatment in different cell types by using irGSEA analysis with the *Ucell* algorithm. B Box plots show the comparisons of irGSEA density of ambient RNA markers before and after decontamination in different cell types. ****P < 0.0001. **Figure S15.** The assessment of the ambient RNAs of the BCAS2 sample in the non-neuronal groups before and after decontamination treatment. MG/Immu, microglia and other immune cell; Ast, astrocyte; Ol, oligodendrocyte; OPC, oligodendrocyte precursor cell; FB, fibroblast; EndoOther, endothelial cell and other cell. A t-SNE plots show the enrichment of ambient RNA markers before and after decontamination treatment in different cell types by using irGSEA analysis with the *Ucell* algorithm. B Box plots show the comparisons of irGSEA density of ambient RNA markers before and after decontamination in different cell types. ****P < 0.0001. **Figure S16.** The analysis of the features of 141 newly discovered genes in Fig. 5E. A Dot plots show the pathways enrichment of 141 newly discovered genes in Fig. 5E. B Volcano plot shows the positions of 141 newly discovered genes before ambient RNAs removal. C Volcano plot shows the positions of 141 newly discovered genes after ambient RNAs removal. **Figure S17.** Pathways enrichment analysis of different MG/Immu subsets. MG1, microglia 1; MG2, microglia 2; *Apoe*^+^ MG/Mac: *Apoe*^+^ microglia/macrophage; Mac2: macrophage; DC, dendritic cell; Other, other nuclei. A-F Dot plots show the pathways enrichment of characteristic genes in different MG/Immu subsets. **Figure S18.** Subgroup analysis of MG/Immu of sham/BCAS mice at single-nuclei resolution before ambient RNAs removal. A-C t-SNE plots show all single-nuclei of microglia in all samples. D Stacked bar plots show the cell proportion of different subgroups annotated after ambient RNAs removal in the NoRemove. C t-SNE plots show all single-nuclei of microglia in the sham and BCAS groups, respectively. E Dot plots show the average expression of specific genes in different subgroups of MG/Immu before ambient RNAs removal. **Figure S19.** Pathways enrichment analysis of different MG/Immu subsets before ambient RNAs removal. **Figure S20.** Pathways enrichment analysis of different MG/Immu subsets before ambient RNAs removal. **Figure S21.** Pathways enrichment analysis of DEGs of specific subgroups between the BCAS and sham groups before ambient RNAs removal. **Figure S22.** The analysis of the difference of MG/Immu subsets between the BCAS and sham groups. A-D Dot plots show the pathways enrichment of characteristic genes in different microglia/immune subsets. E Venn plots show the overlapping genes between two corresponding subgroups from the NoRemove and DeContam, respectively. F Box plots show the comparisons of LogFC of shared genes in panel E between two corresponding subgroups from the NoRemove and DeContam, respectively. ****P < 0.0001. **Figure S23.** The comparisons of the ranking of *Apoe* before and after ambient RNAs removal.

**Additional file 2: Table S1.** The information for details of the nuclei suspension of four different samples. **Table S2.** The information for details of the reverse transcription products and snRNA-seq libraries of four different samples. **Table S3.** The differentially expressed genes list of the MG/Immu subset before decontamination by using muscat.. **Table S4.** The differentially expressed genes list of the MG/Immu subset after decontamination by using muscat. **Table S5.** The differentially expressed genes list of the cortex-specific transcriptomeby using DEseq2.
