# Supplementary material for: Ambient RNAs removal of cortex-specific snRNA-seq reveals Apoe+ microglia/macrophage after deeper cerebral hypoperfusion in mice
Source: J Neuroinflammation. 2023 Jun 26;20:152. doi: 10.1186/s12974-023-02831-9 (PMC10294316; doi:10.1186/s12974-023-02831-9)
Supplement: Supplementary file 1 — Additional file 1: Figure S1. Microscopic examinations of the nuclei suspension of the sham1 sample. A Microscopic image in different channels. FL1 showing live cells, FL2 showing dead cells. B Bar plots showing the number of nuclei in different sizes according to the images of bright field. C, D Bar plots showing the number of nuclei in different RFUs of FL1and FL2, respectively. RFU, relative fluorescence units. Figure S2. Microscopic examinations of the nuclei suspension of the BCAS1 sample. A Microscopic image in different channels. FL1 showing live cells, FL2 showing dead cells. B Bar plots showing the number of nuclei in different sizes according to the images of bright field. C, D Bar plots showing the number of nuclei in different RFUs of FL1and FL2, respectively. RFU, relative fluorescence units. Figure S3. Microscopic examinations of the nuclei suspension of the sham2 sample. A Microscopic image in different channels. FL1 showing live cells, FL2 showing dead cells. B Bar plots showing the number of nuclei in different sizes according to the images of bright field. C, D Bar plots showing the number of nuclei in different RFUs of FL1and FL2, respectively. RFU, relative fluorescence units. Figure S4. Microscopic examinations of the nuclei suspension of the BCAS2 sample. A Microscopic image in different channels. FL1 showing live cells, FL2 showing dead cells. B Bar plots showing the number of nuclei in different sizes according to the images of bright field. C, D Bar plots showing the number of nuclei in different RFUs of FL1and FL2, respectively. RFU, relative fluorescence units. Figure S5. Gel electrophoresis of different samples in different lanes as measured by an Agilent 4200. C1, the cDNA products of sham1; C2, the cDNA products of BCAS1; C3, the cDNA products of sham2; C4, the cDNA products of BCAS2; C5, the snRNA-seq library of sham1; C6, the snRNA-seq library of BCAS1; C7, the snRNA-seq library of sham2; C8, the snRNA-seq library of BCAS2. Figure S6. [file 12974_2023_2831_MOESM1_ESM.docx]

**
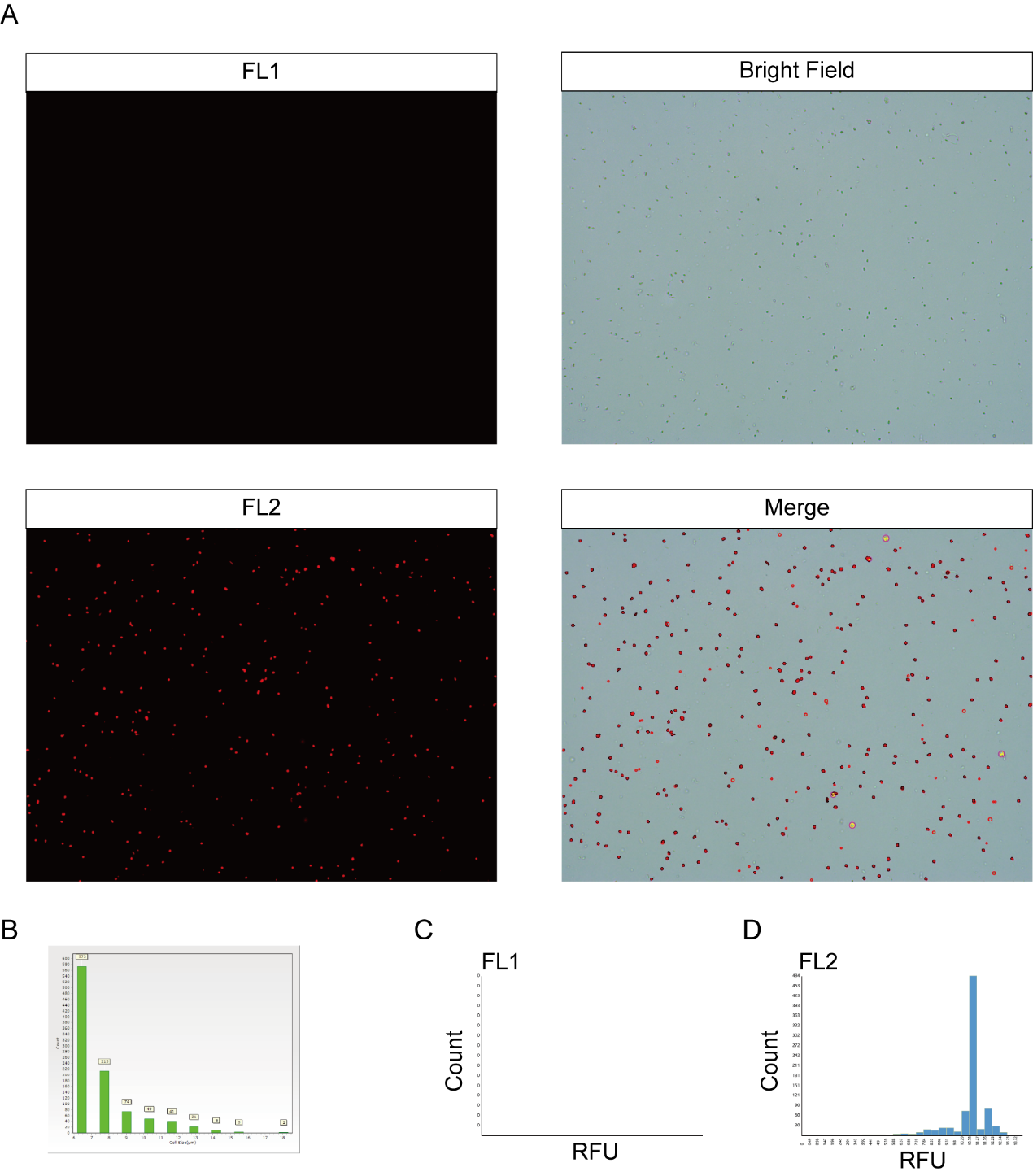
**

**Figure S1.** Microscopic examinations of the nuclei suspension of the sham1 sample. **A** Microscopic image in different channels. FL1 showing live cells, FL2 showing dead cells. **B** Bar plots showing the number of nuclei in different sizes according to the images of bright field. **C, D** Bar plots showing the number of nuclei in different RFUs of FL1 and FL2, respectively. RFU, relative fluorescence units.


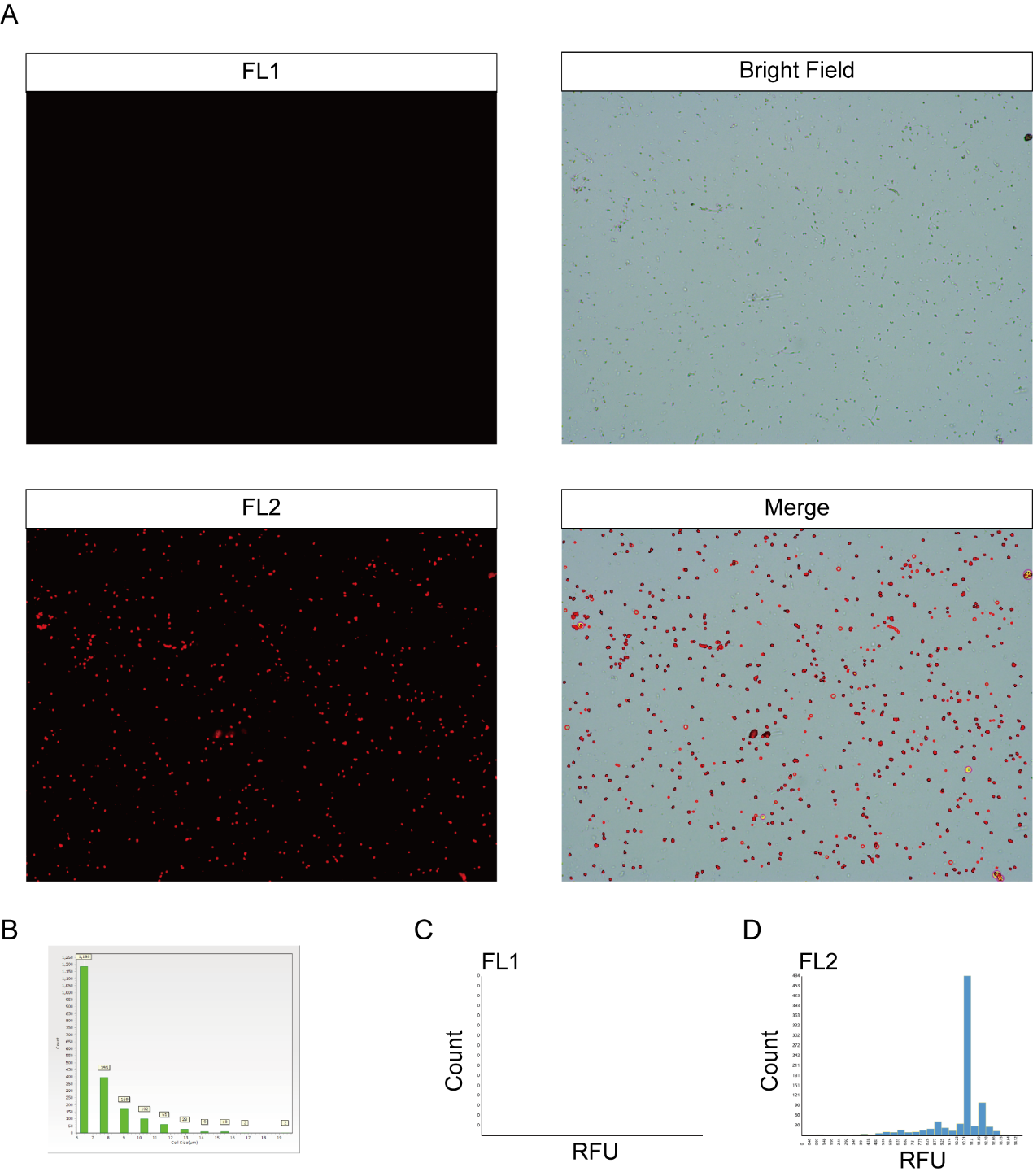


**Figure S2.** Microscopic examinations of the nuclei suspension of the BCAS1 sample. **A** Microscopic image in different channels. FL1 showing live cells, FL2 showing dead cells. **B** Bar plots showing the number of nuclei in different sizes according to the images of bright field. **C, D** Bar plots showing the number of nuclei in different RFUs of FL1 and FL2, respectively. RFU, relative fluorescence units.


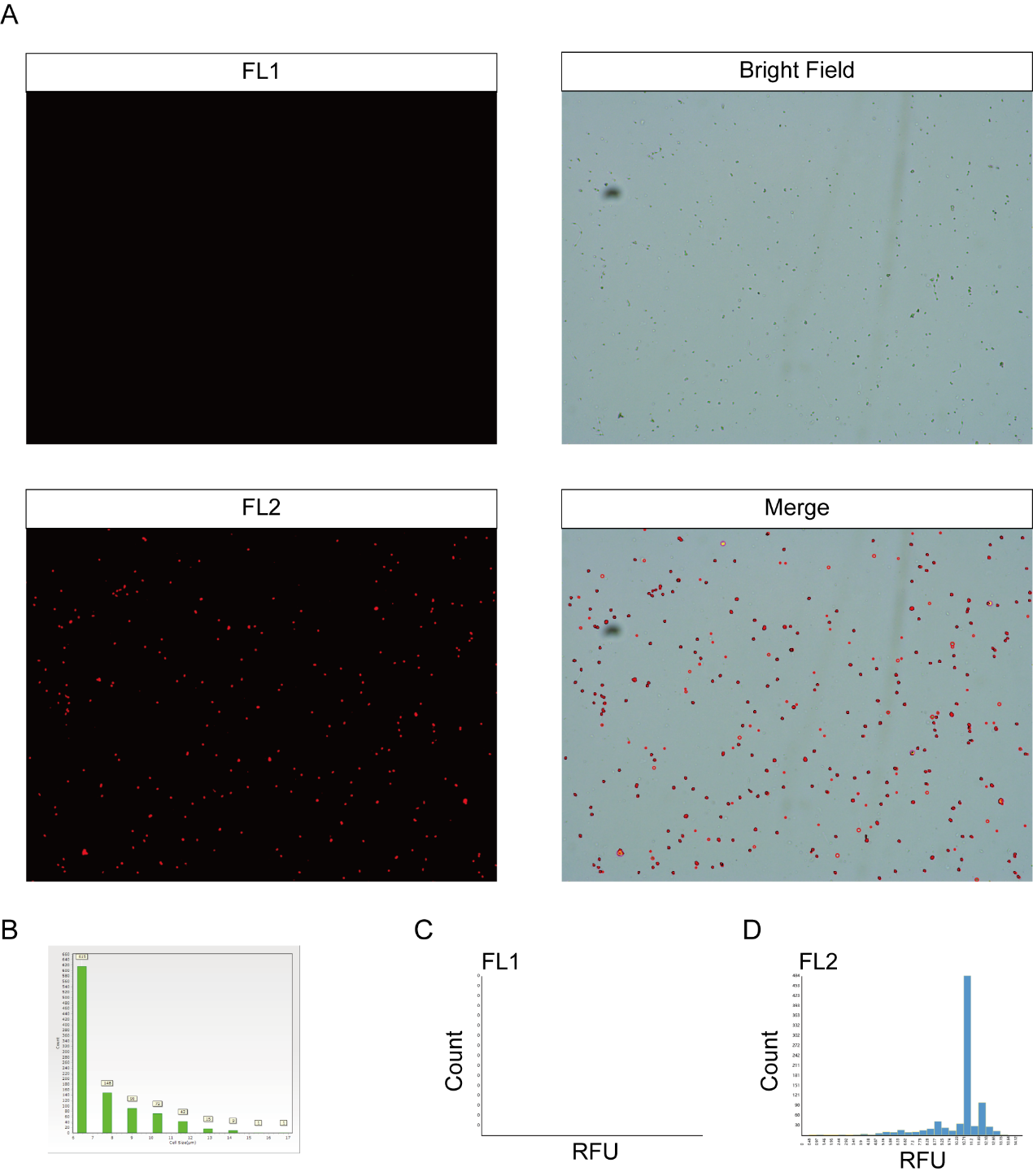


**Figure S3.** Microscopic examinations of the nuclei suspension of the sham2 sample. **A** Microscopic image in different channels. FL1 showing live cells, FL2 showing dead cells. **B** Bar plots showing the number of nuclei in different sizes according to the images of bright field. **C, D** Bar plots showing the number of nuclei in different RFUs of FL1 and FL2, respectively. RFU, relative fluorescence units.


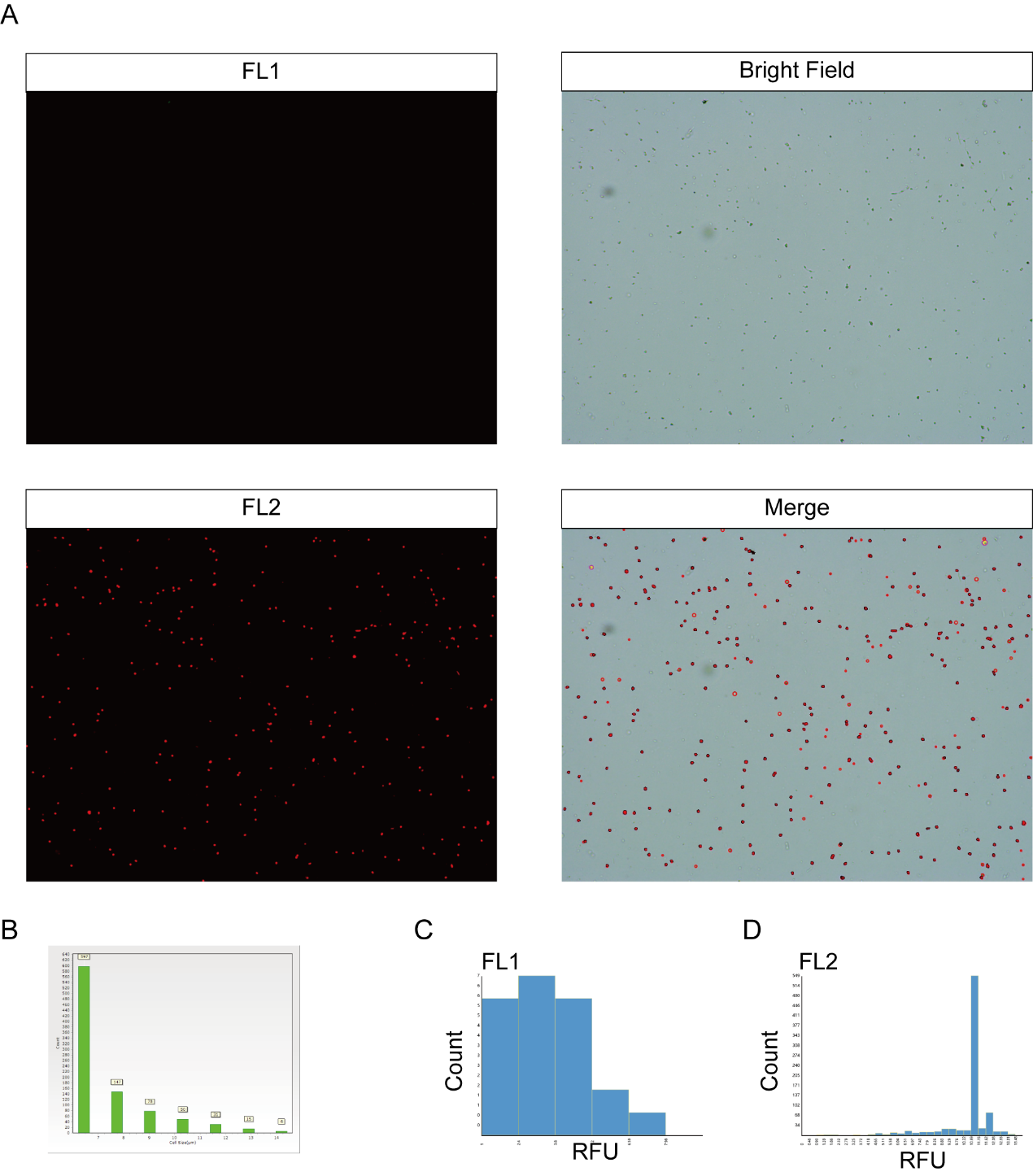


**Figure S4.** Microscopic examinations of the nuclei suspension of the BCAS2 sample. **A** Microscopic image in different channels. FL1 showing live cells, FL2 showing dead cells. **B** Bar plots showing the number of nuclei in different sizes according to the images of bright field. **C, D** Bar plots showing the number of nuclei in different RFUs of FL1 and FL2, respectively. RFU, relative fluorescence units.

**
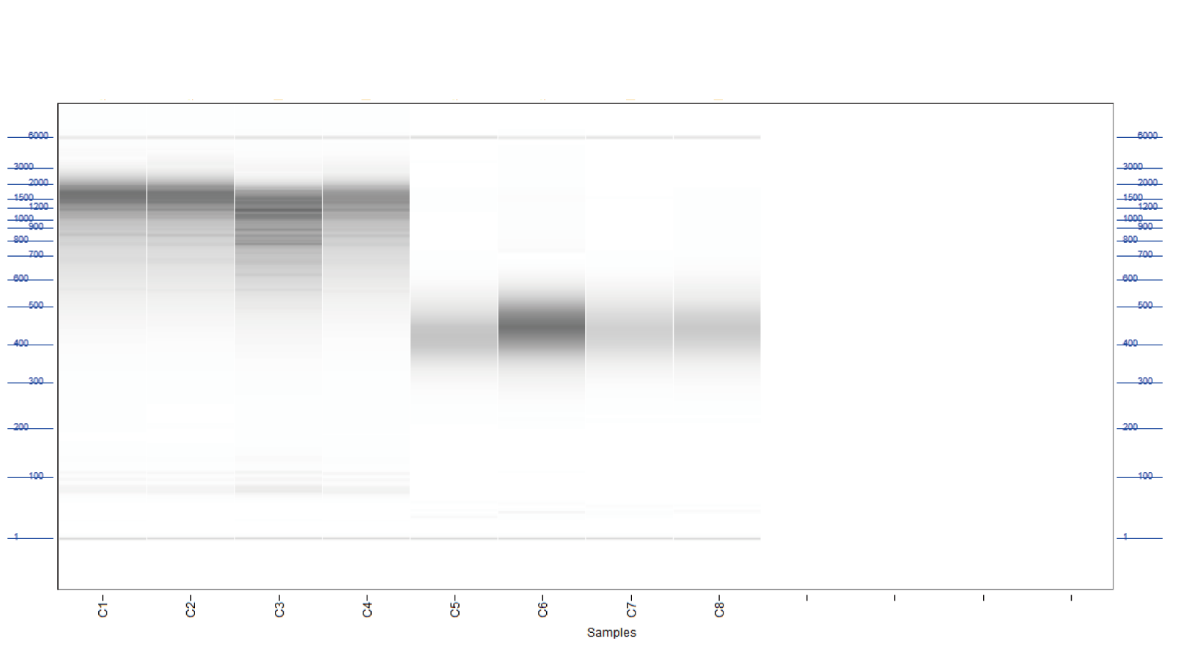
**

**Figure S5.** Gel electrophoresis of different samples in different lanes (C1-C8) as measured by an Agilent 4200. C1, the cDNA products of sham1; C2, the cDNA products of BCAS1; C3, the cDNA products of sham2; C4, the cDNA products of BCAS2; C5, the snRNA-seq library of sham1; C6, the snRNA-seq library of BCAS1; C7, the snRNA-seq library of sham2; C8, the snRNA-seq library of BCAS2.


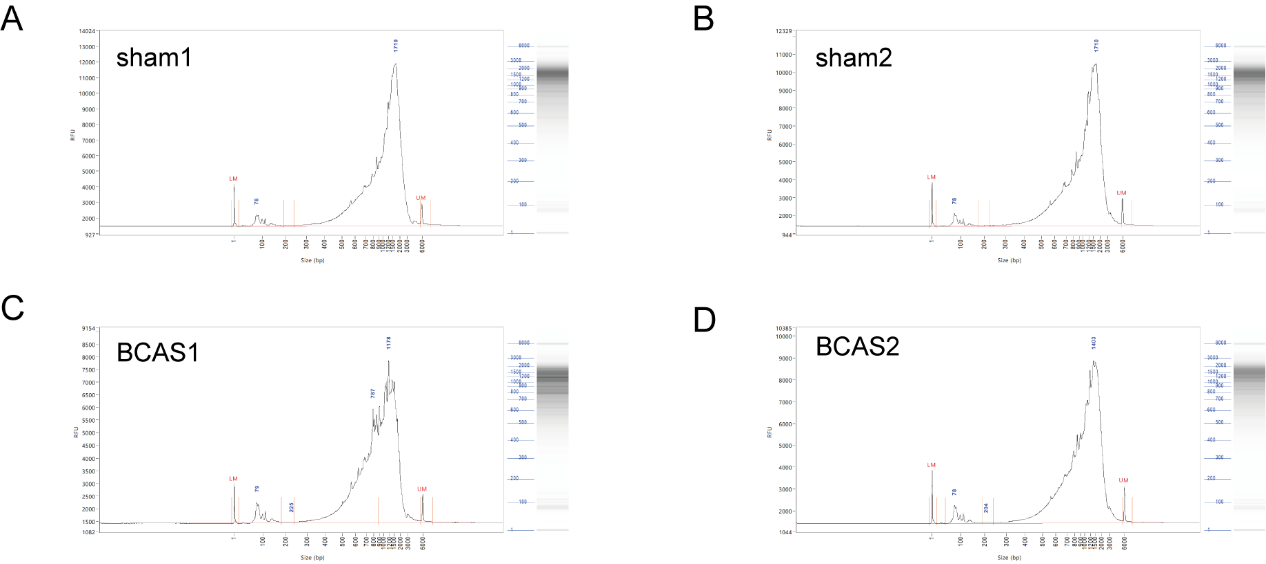


**Figure S6.** The reverse transcription products fragment sizes of different samples as measured by an Agilent 4200.

**
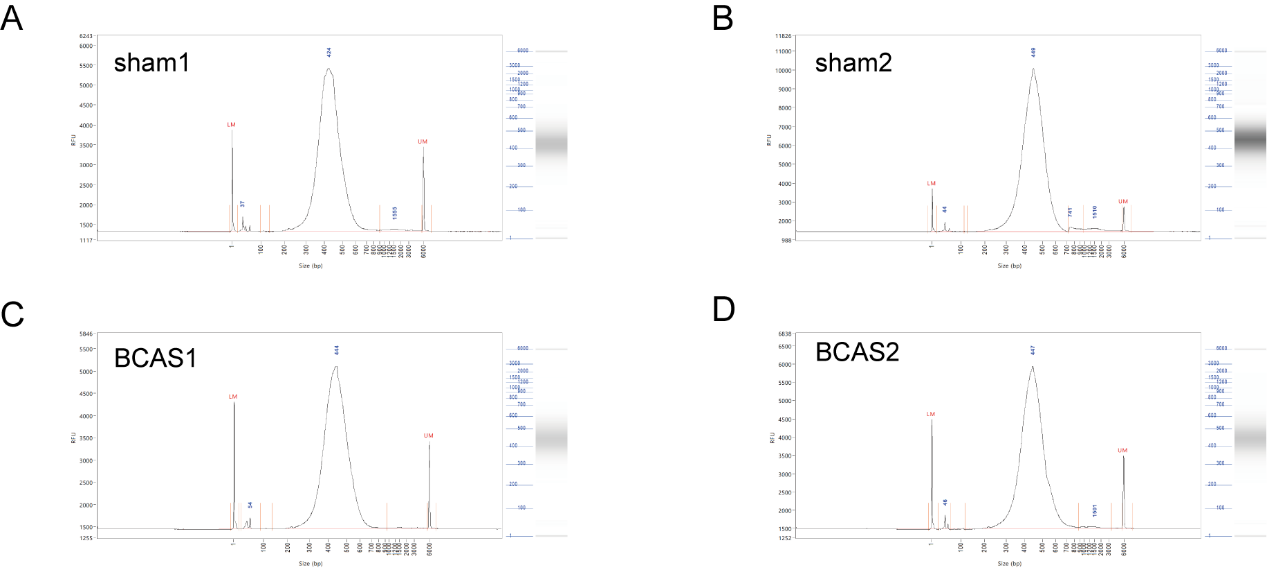
**

**Figure S7.** The snRNA-seq libraries fragment sizes of different samples as measured by an Agilent 4200.

**
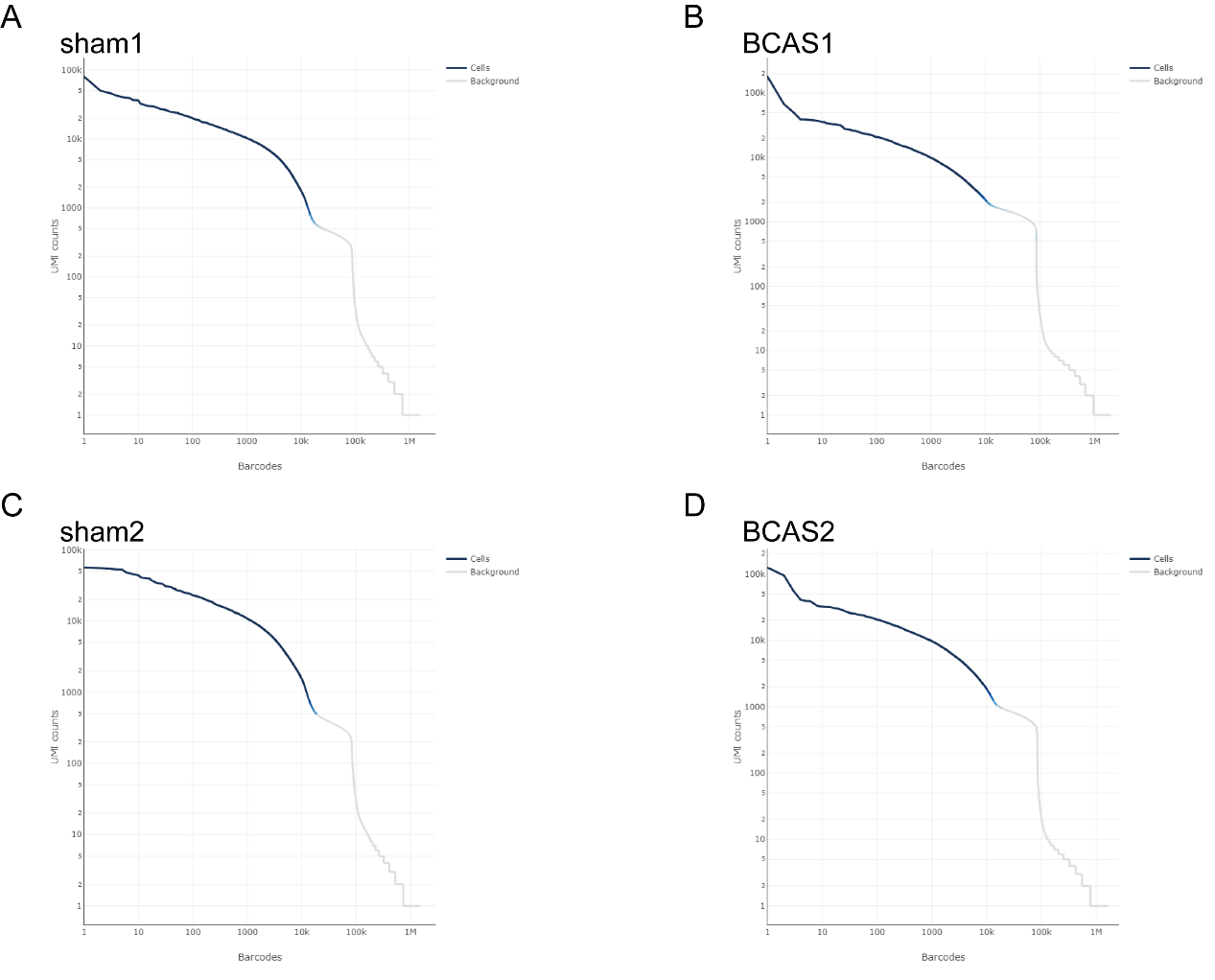
**

**Figure S8.** The barcode rank plots of four different samples generated by the software CellRanger.

**Figure S9.** The expression of microglia-specific gene in the sham and BCAS group. **A** t-SNE plots show the expression of *Hexb* in different groups between the sham and BCAS groups.**
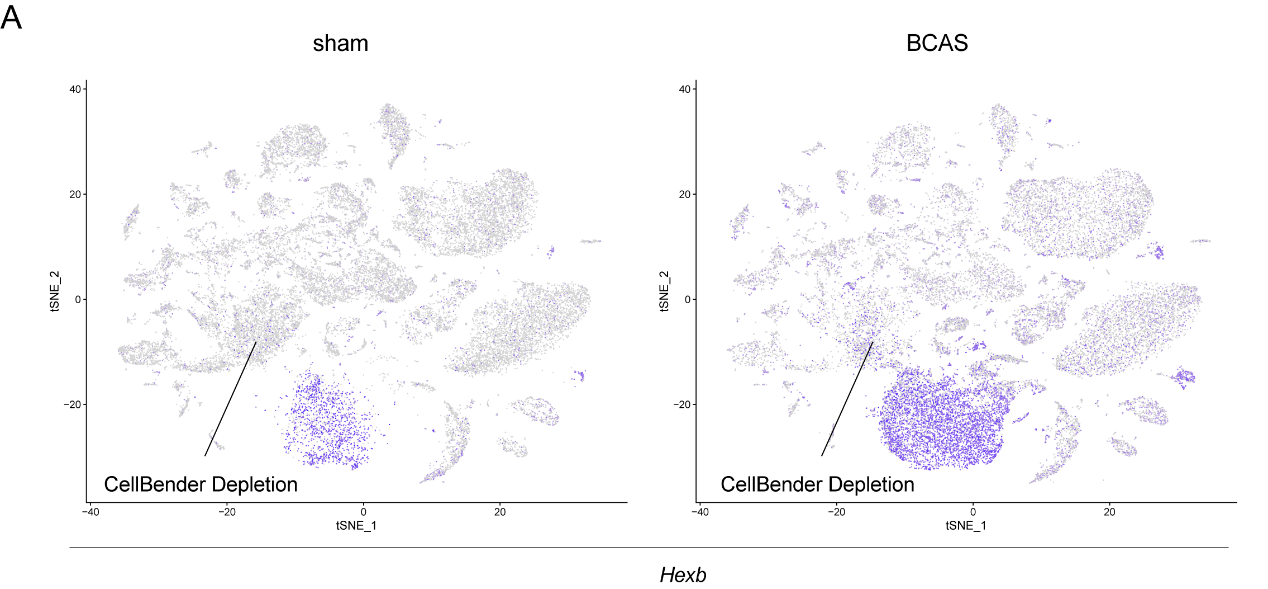
**

**Figure S10.**
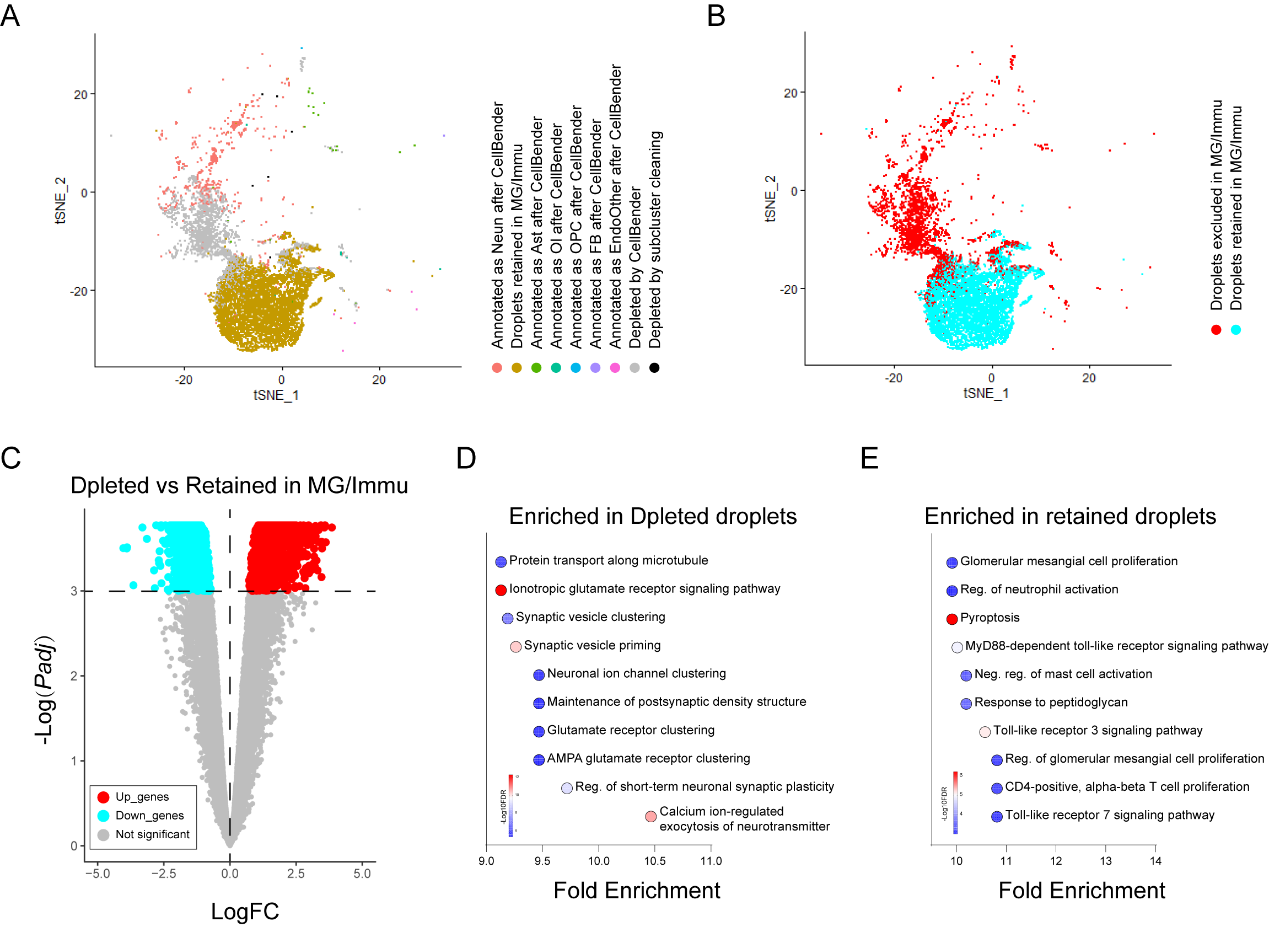
The analysis of the features of the droplets depleted from MG/Immu in the BCAS group. **A, B** T-SNE plot shows single-nuclei in all droplets in the MG/Immu of the BCAS group before ambient RNAs removal. **C** Volcano plot shows DEGs of MG/Immu between the depleted and retained droplets. **D, E** Dot plots show the pathways enrichment of the genes enriched in depleted and retained droplets, respectively.


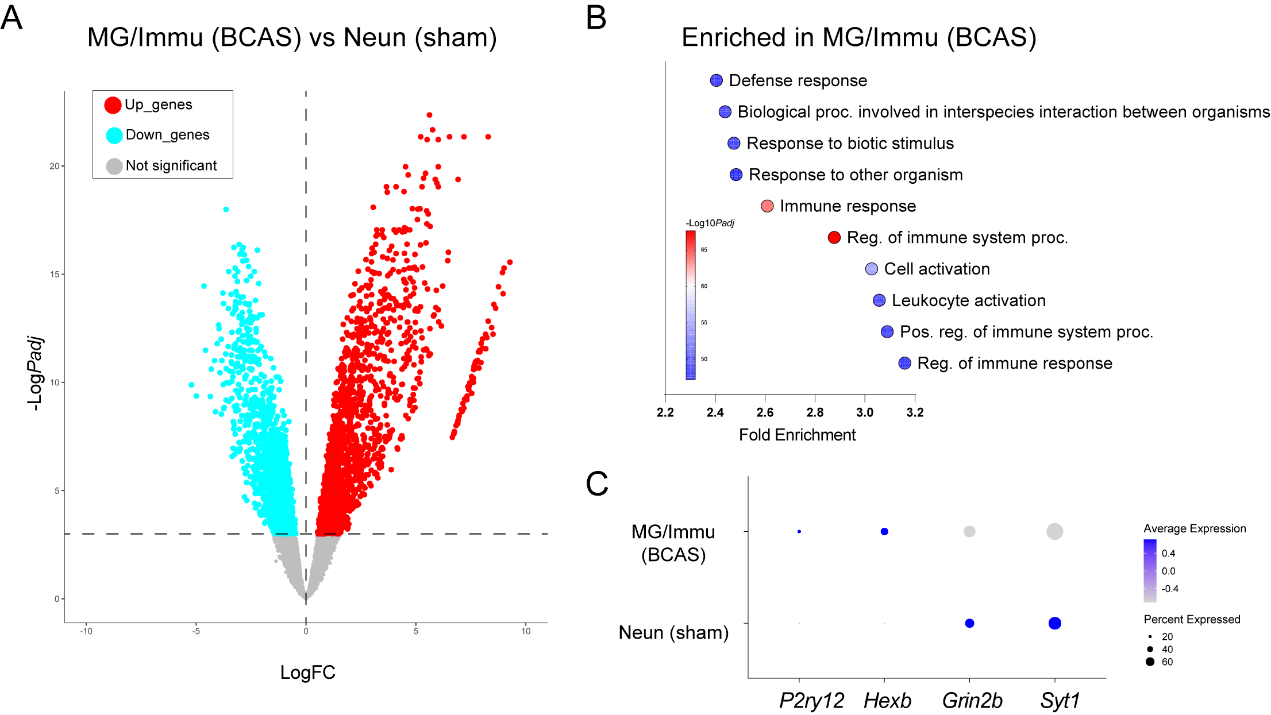


**Figure S11.** The analysis of the features of the droplets depleted from MG/Immu in the BCAS group. **A** Volcano plot shows DEGs of the droplets depleted by CellBender between the BCAS and sham groups. **B** Dot plots show the pathways enrichment of up-genes enriched in panel **A**. **C** Dot plots show the average expression of specific genes in the droplets depleted by CellBender between the BCAS and sham groups.

**
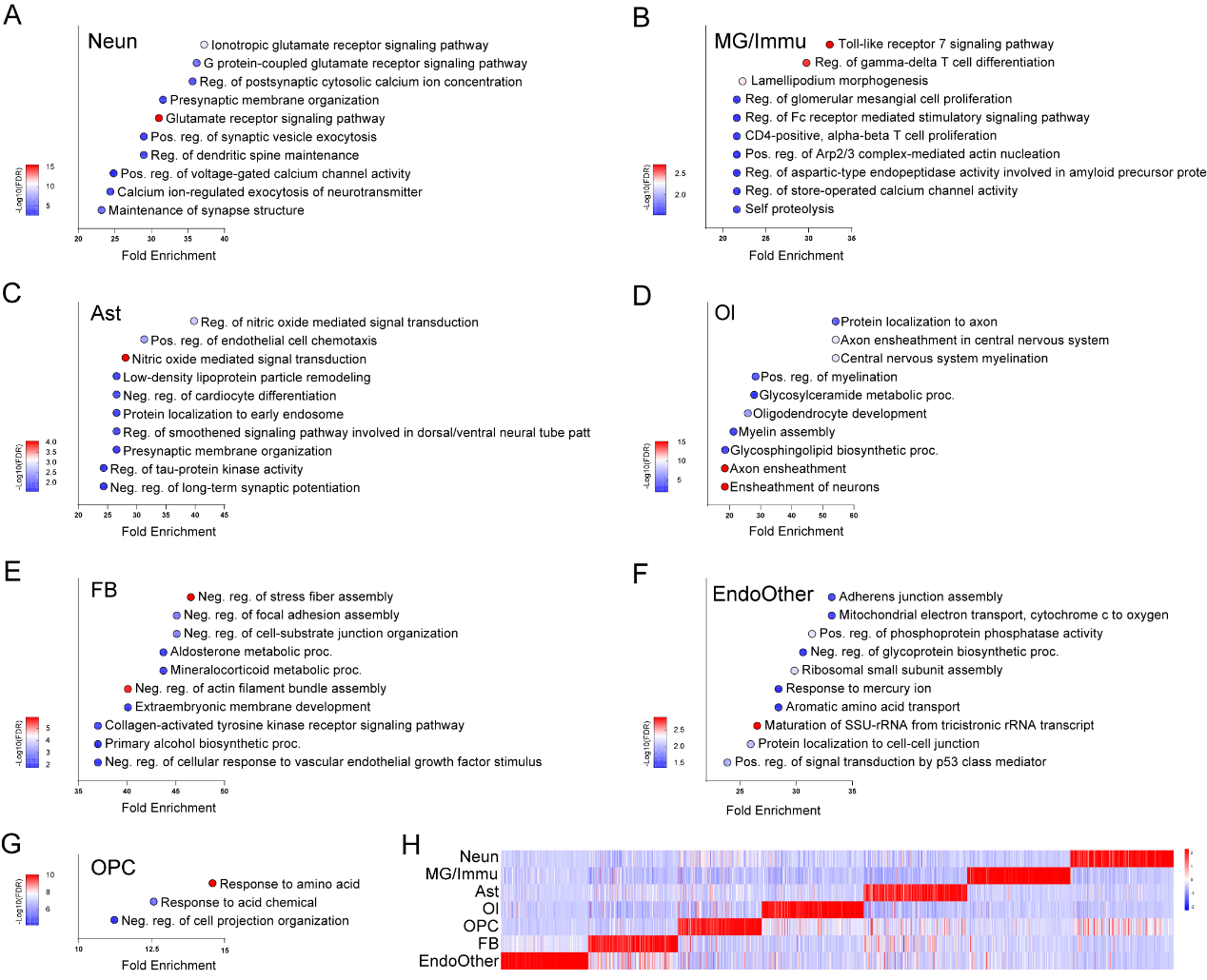
**

**Figure S12.** Pathways enrichment analysis of different cell types. Neun, neuron; MG/Immu, microglia and other immune cell; Ast, astrocyte; Ol, oligodendrocyte; OPC, oligodendrocyte precursor cell; FB, fibroblast; EndoOther, endothelial cell and other cell. **A-G** Dot plots show the pathways enrichment of top 200 characteristic genes in different cell types. **H** Heatmap shows the expression of top 200 characteristic genes in different cell types.


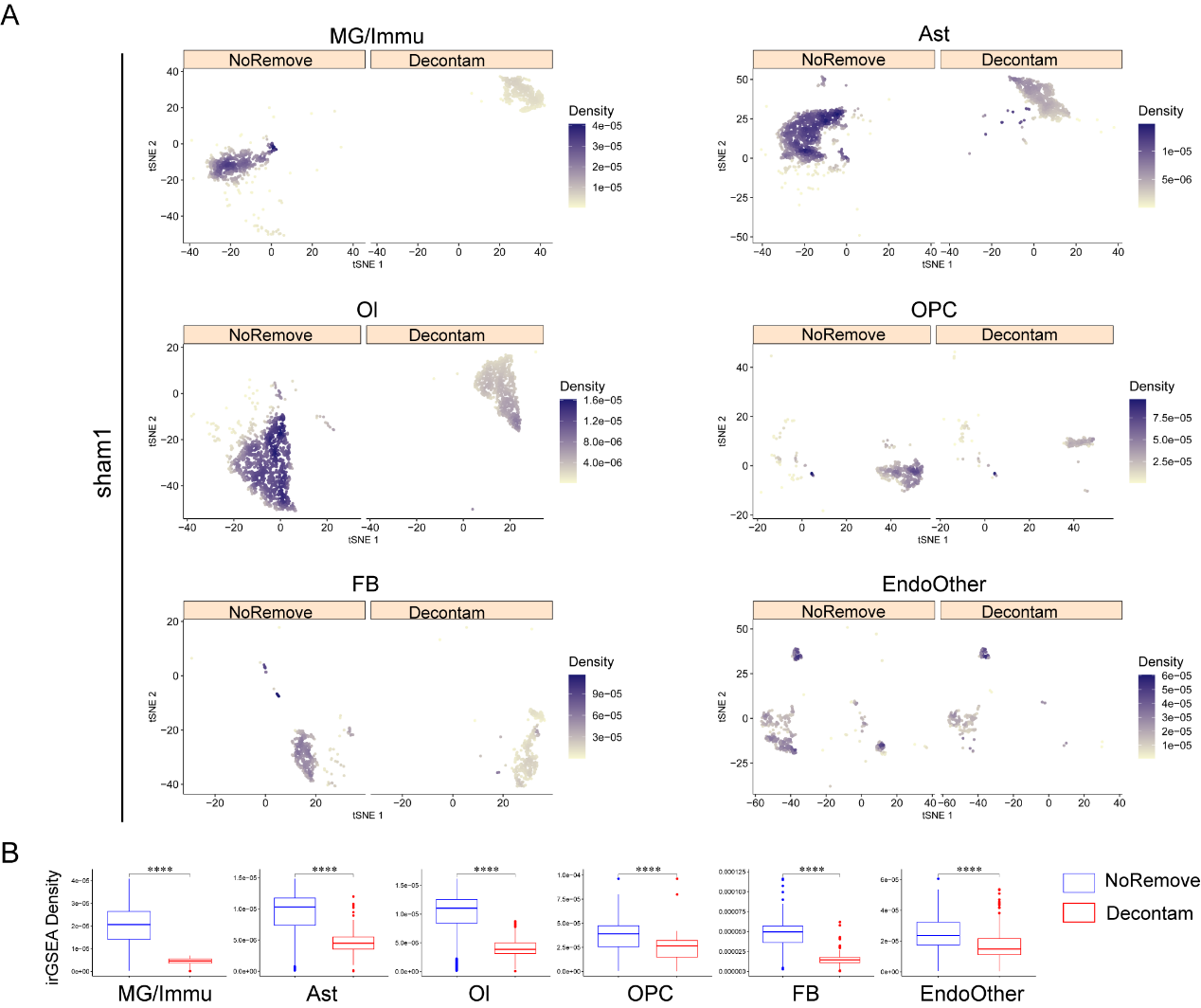


**Figure S13.** The assessment of the ambient RNAs of the sham1 sample in the non-neuronal groups before and after decontamination treatment. MG/Immu, microglia and other immune cell; Ast, astrocyte; Ol, oligodendrocyte; OPC, oligodendrocyte precursor cell; FB, fibroblast; EndoOther, endothelial cell and other cell. **A** t-SNE plots show the enrichment of ambient RNA markers before and after decontamination treatment in different cell types by using irGSEA analysis with the *Ucell* algorithm. **B** Box plots show the comparisons of irGSEA density of ambient RNA markers before and after decontamination in different cell types. *****P* < 0.0001.


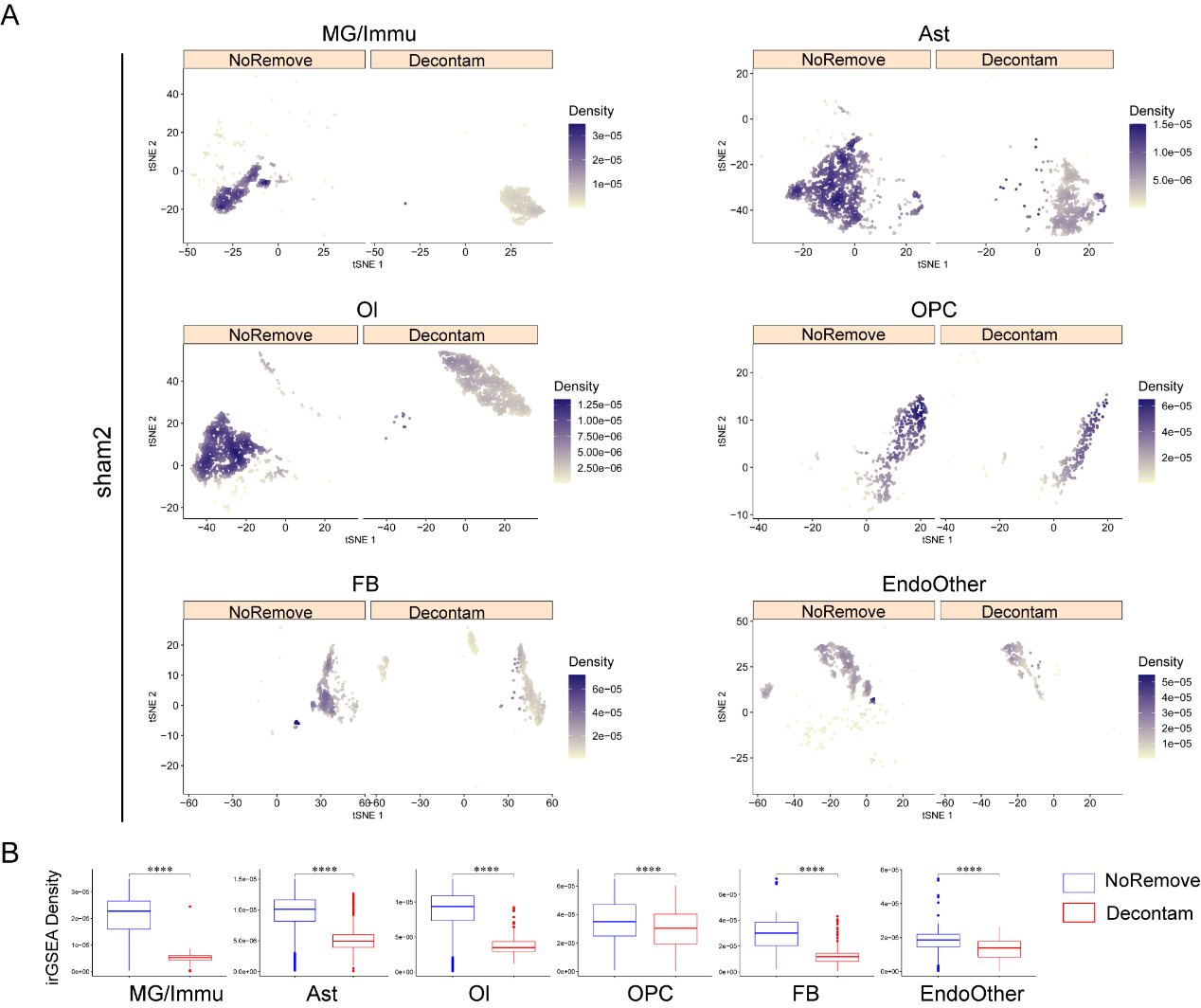


**Figure S14.** The assessment of the ambient RNAs of the sham2 sample in the non-neuronal groups before and after decontamination treatment. MG/Immu, microglia and other immune cell; Ast, astrocyte; Ol, oligodendrocyte; OPC, oligodendrocyte precursor cell; FB, fibroblast; EndoOther, endothelial cell and other cell. **A** t-SNE plots show the enrichment of ambient RNA markers before and after decontamination treatment in different cell types by using irGSEA analysis with the *Ucell* algorithm. **B** Box plots show the comparisons of irGSEA density of ambient RNA markers before and after decontamination in different cell types. *****P* < 0.0001.


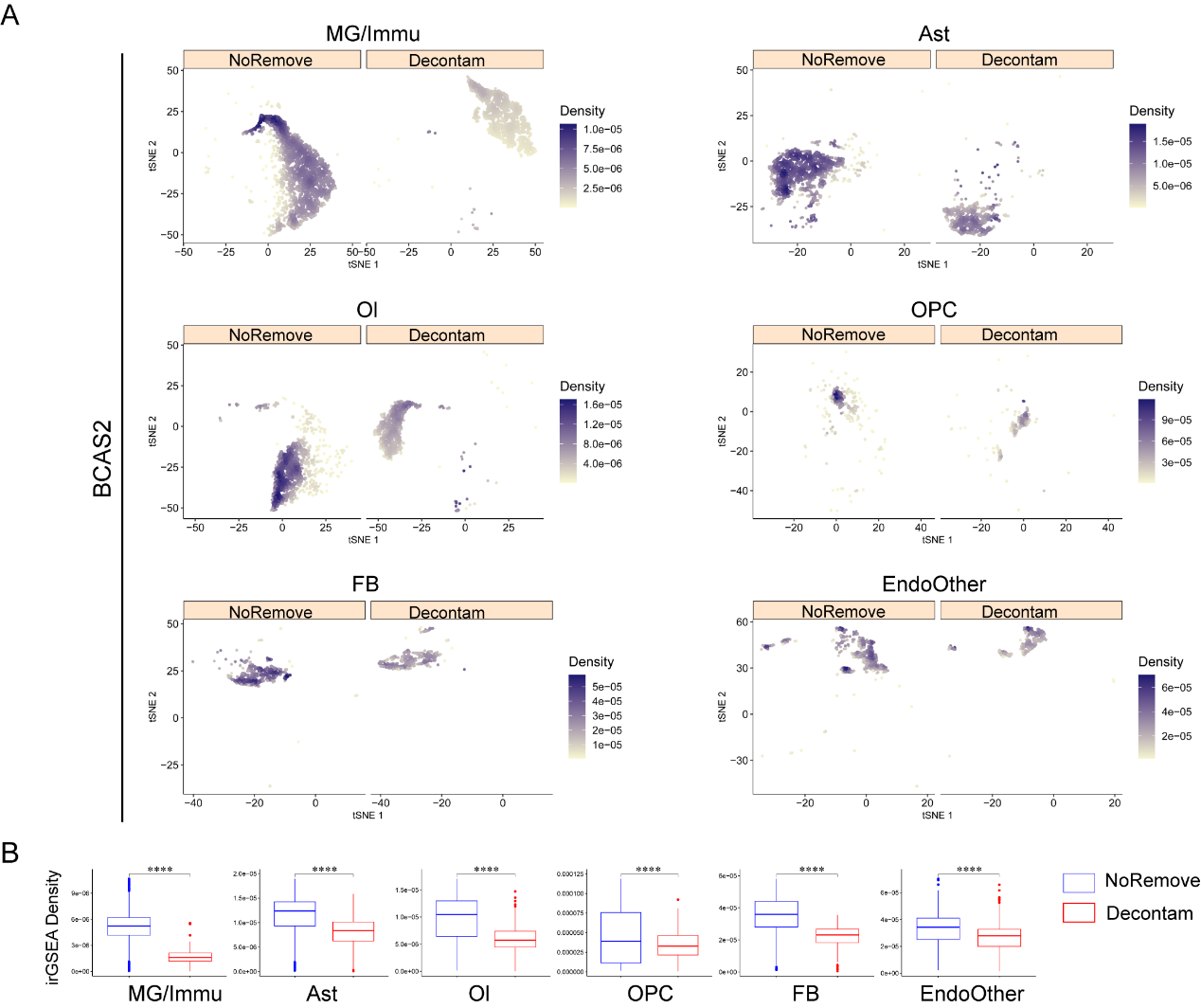


**Figure S15.** The assessment of the ambient RNAs of the BCAS2 sample in the non-neuronal groups before and after decontamination treatment. MG/Immu, microglia and other immune cell; Ast, astrocyte; Ol, oligodendrocyte; OPC, oligodendrocyte precursor cell; FB, fibroblast; EndoOther, endothelial cell and other cell. **A** t-SNE plots show the enrichment of ambient RNA markers before and after decontamination treatment in different cell types by using irGSEA analysis with the *Ucell* algorithm. **B** Box plots show the comparisons of irGSEA density of ambient RNA markers before and after decontamination in different cell types. *****P* < 0.0001.


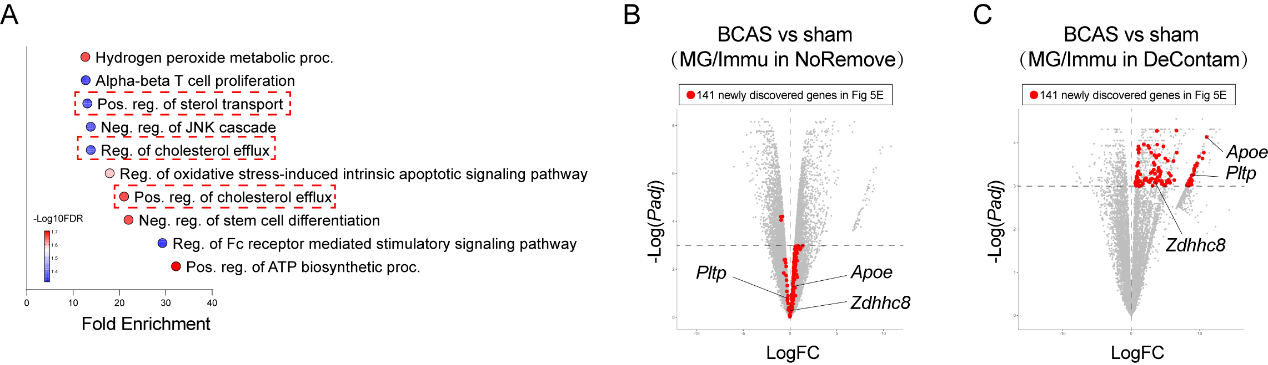
**Figure S16.** The analysis of the features of 141 newly discovered genes in **Fig. 5E**. **A** Dot plots show the pathways enrichment of 141 newly discovered genes in **Fig. 5E**. **B** Volcano plot shows the positions of 141 newly discovered genes before ambient RNAs removal. **C** Volcano plot shows the positions of 141 newly discovered genes after ambient RNAs removal.


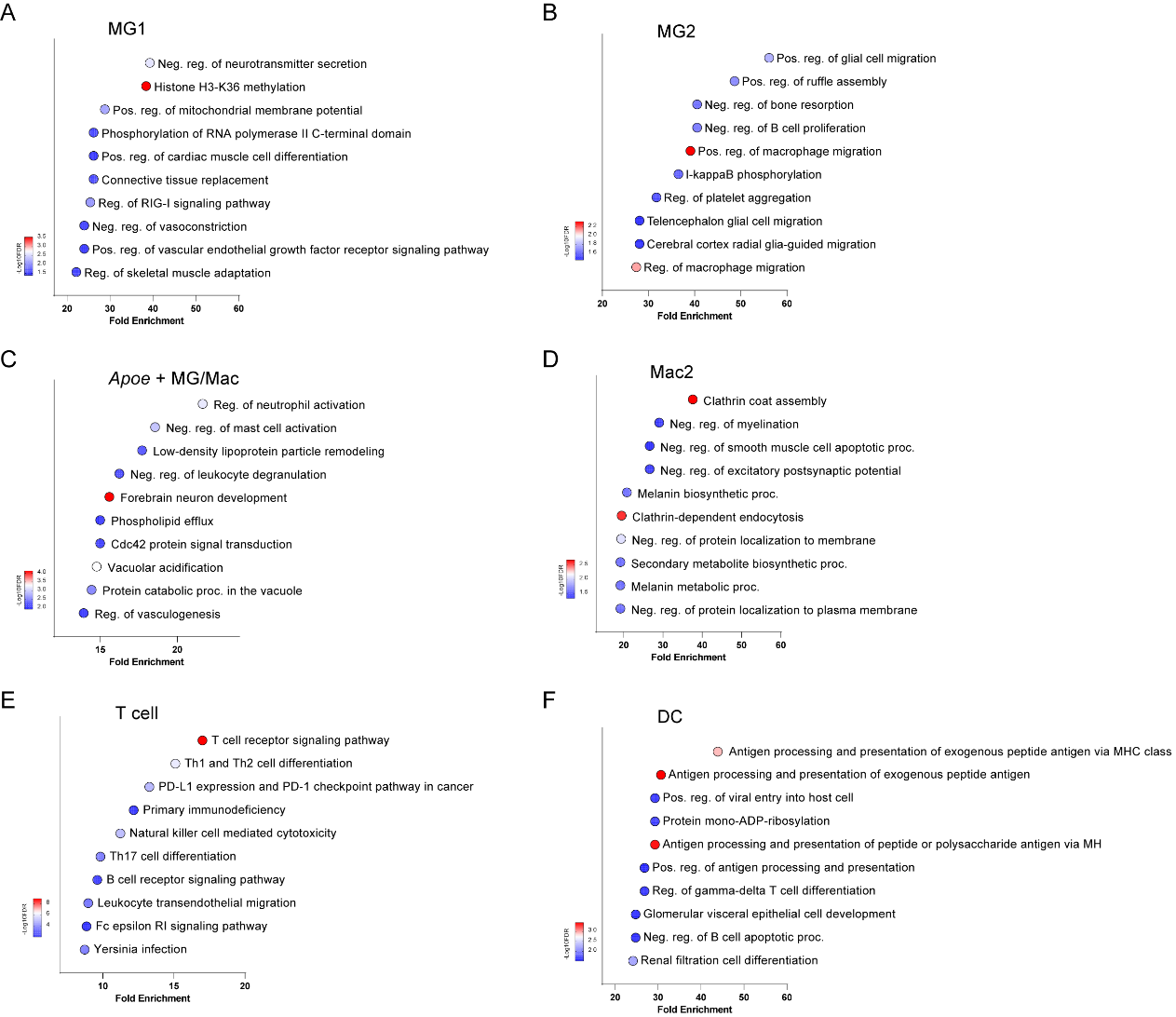


**Figure S17.** Pathways enrichment analysis of different MG/Immu subsets. MG1, microglia 1; MG2, microglia 2; *Apoe*^+^ MG/Mac: *Apoe*^+^ microglia/macrophage; Mac2: macrophage; DC, dendritic cell; Other, other nuclei. **A-F** Dot plots show the pathways enrichment of characteristic genes in different MG/Immu subsets.

**
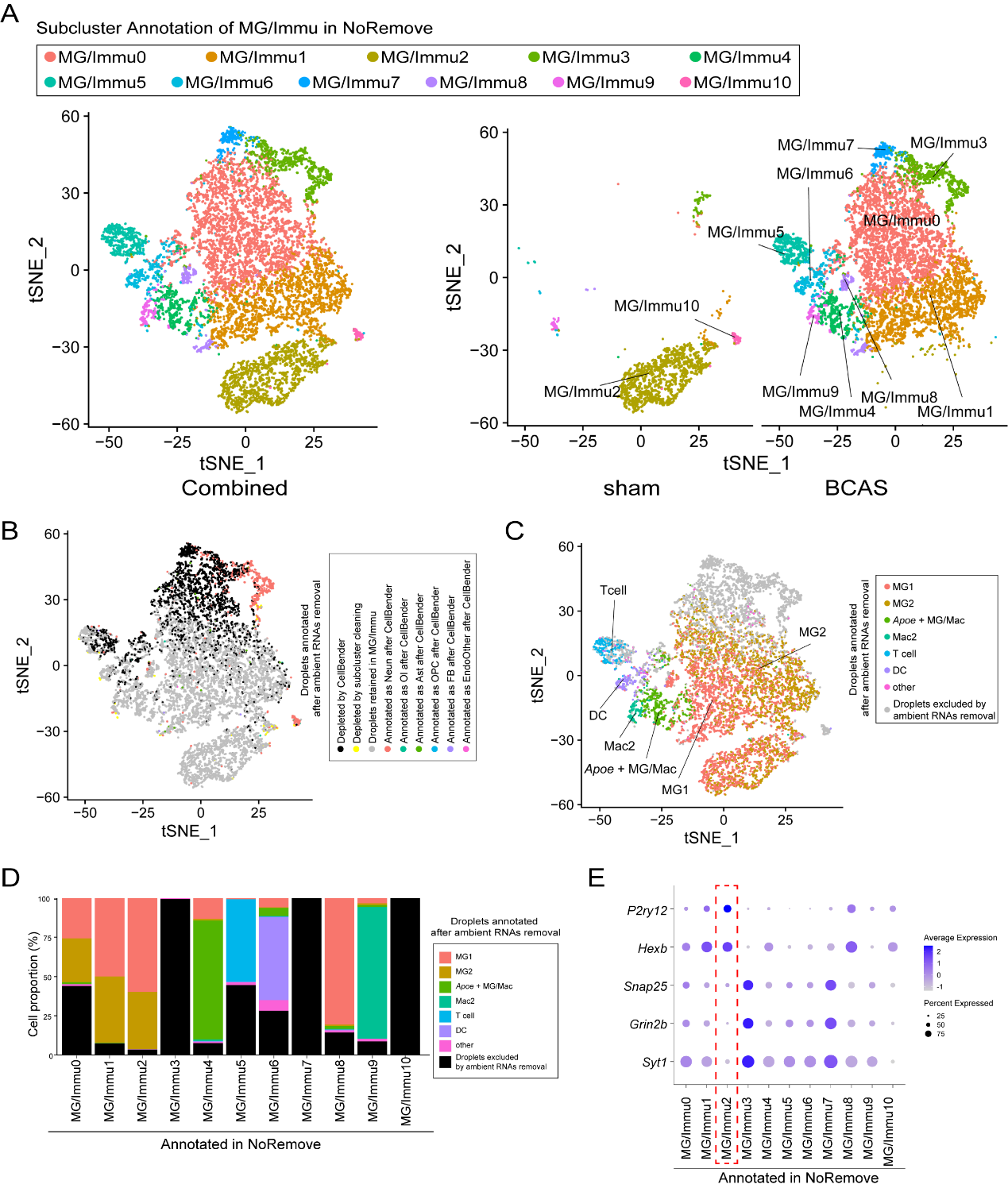
**

**Figure S18.** Subgroup analysis of MG/Immu of sham/BCAS mice at single-nuclei resolution before ambient RNAs removal. **A-C** t-SNE plots show all single-nuclei of microglia in all samples. **D** Stacked bar plots show the cell proportion of different subgroups annotated after ambient RNAs removal in the NoRemove. **C** t-SNE plots show all single-nuclei of microglia in the sham and BCAS groups, respectively. **E** Dot plots show the average expression of specific genes in different subgroups of MG/Immu before ambient RNAs removal.

**
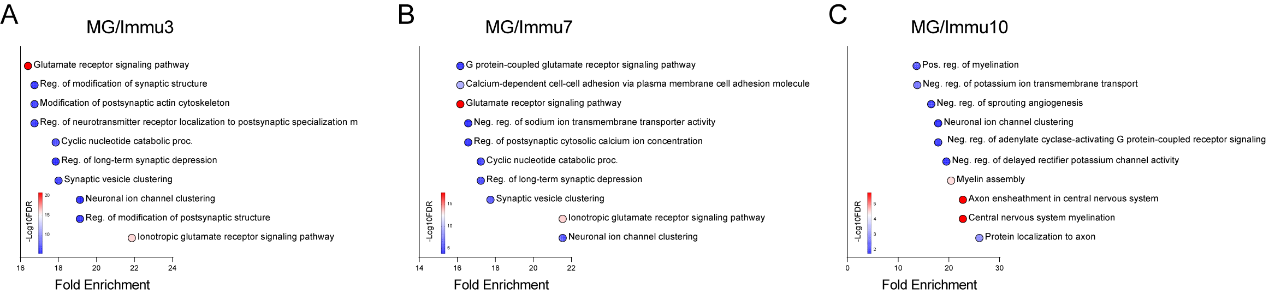
**

**Figure S19.** Pathways enrichment analysis of different MG/Immu subsets before ambient RNAs removal.

**
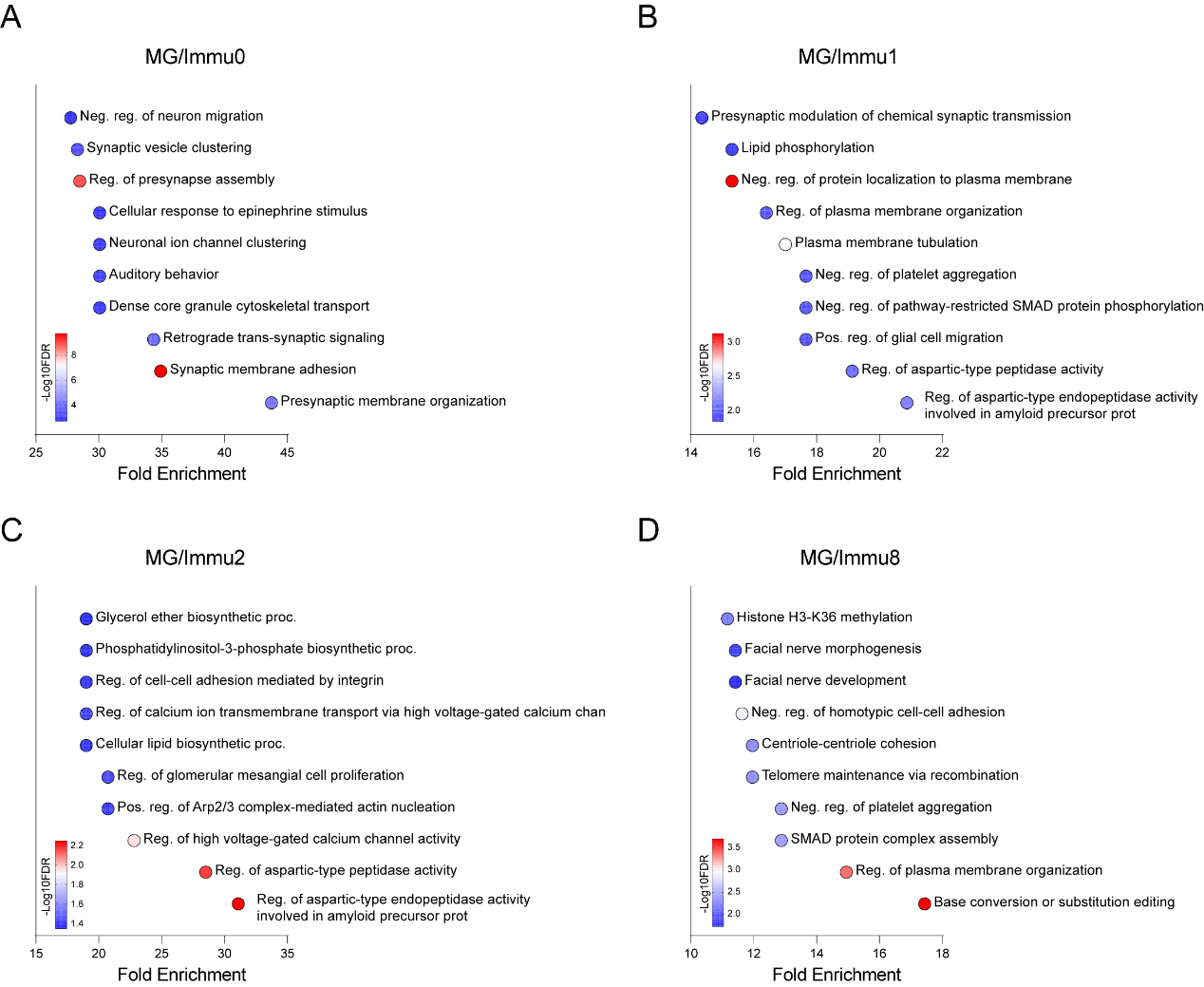
**

**Figure S20.** Pathways enrichment analysis of different MG/Immu subsets before ambient RNAs removal.

**
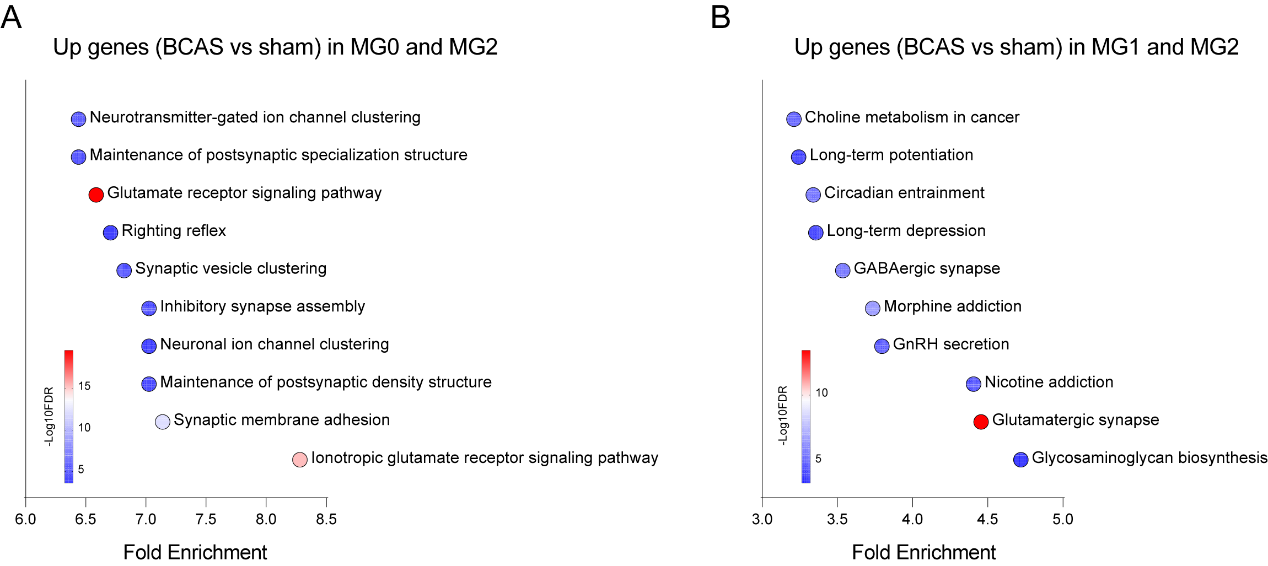
**

**Figure S21.** Pathways enrichment analysis of DEGs of specific subgroups between the BCAS and sham groups before ambient RNAs removal.

**
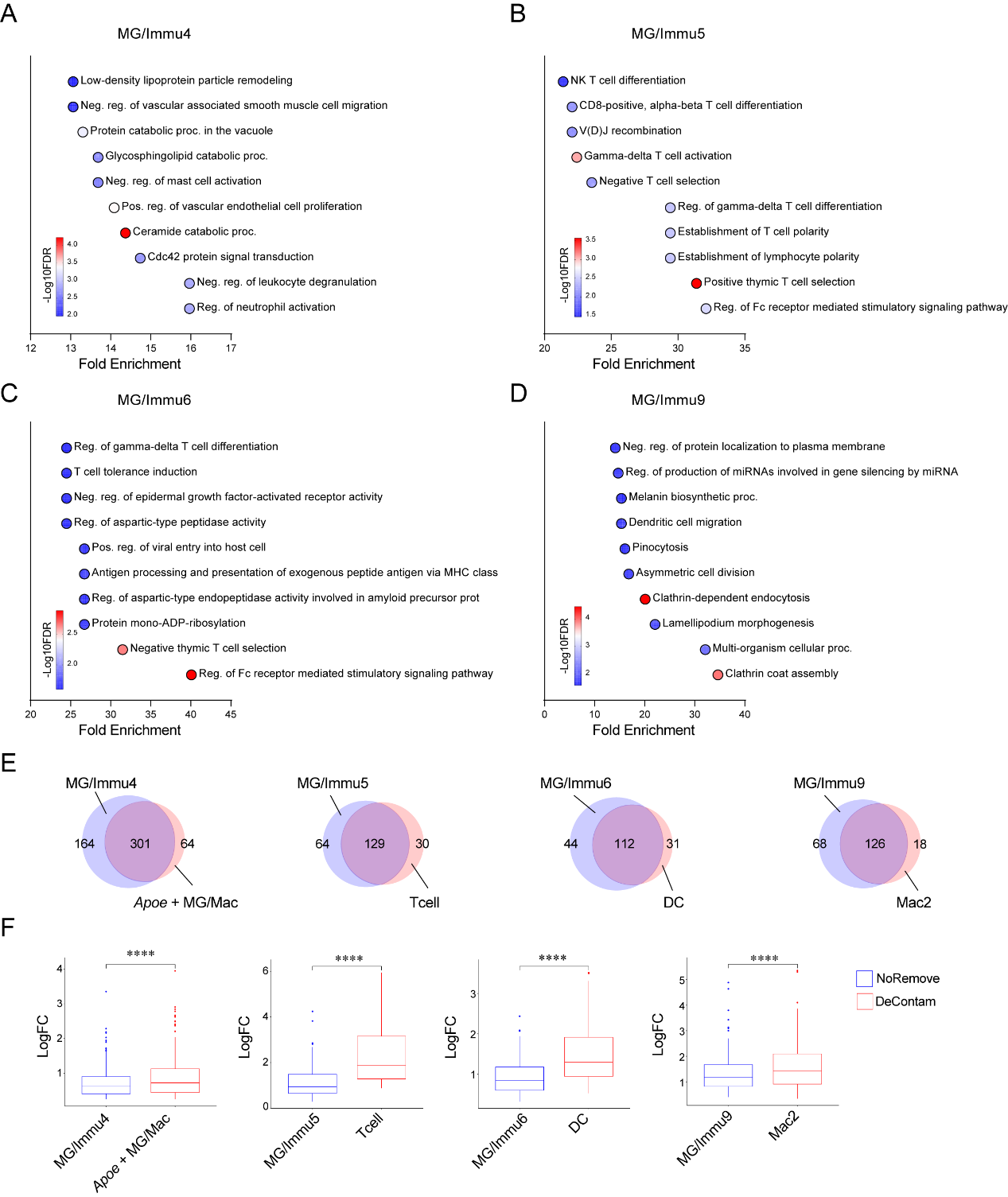
**

**Figure S22.** The analysis of the difference of MG/Immu subsets between the BCAS and sham groups. **A-D** Dot plots show the pathways enrichment of characteristic genes in different microglia/immune subsets. **E** Venn plots show the overlapping genes between two corresponding subgroups from the NoRemove and DeContam, respectively. **F** Box plots show the comparisons of LogFC of shared genes in panel **E** between two corresponding subgroups from the NoRemove and DeContam, respectively. *****P* < 0.0001.

**
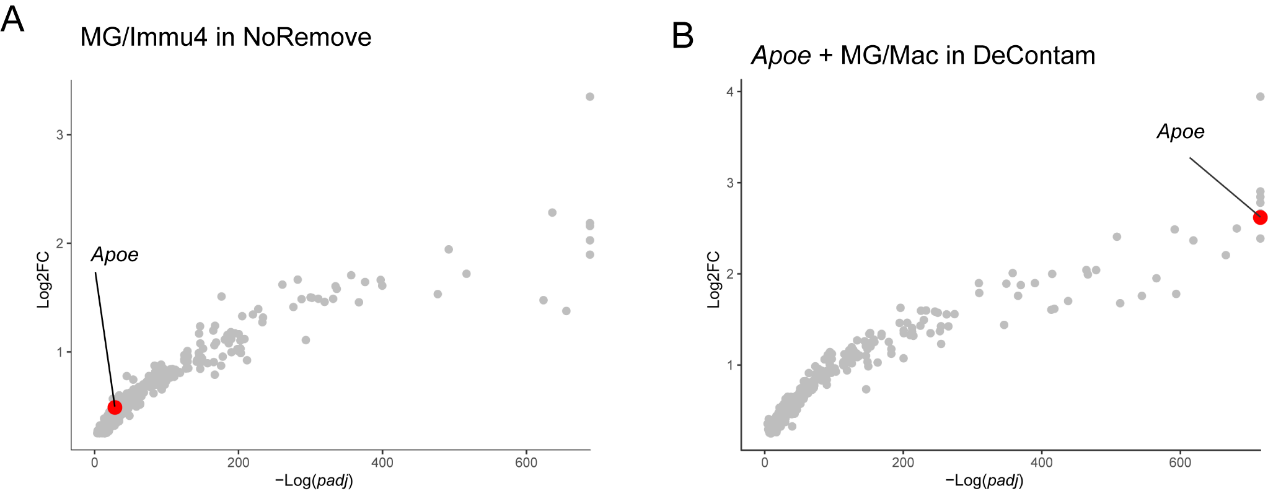
**

**Figure S23.** The comparisons of the ranking of *Apoe* before and after ambient RNAs removal.
